# Exploring the relationship between social activities and financial risk aversion in adults aged 50 + with depression caseness

**DOI:** 10.1186/s13584-024-00621-z

**Published:** 2024-07-30

**Authors:** Shay Musbat, Inbal Reuveni, Racheli Magnezi

**Affiliations:** 1 Department of Management, Health Systems Management Program, Bar-Ilan University, 5290002 Ramat Gan, Israel; 2 Department of Psychiatry, Hadassah Hebrew University Medical Center, 9112001 Ein Kerem, Jerusalem, Israel

**Keywords:** Depression, Financial decisions, Risk aversion, Risk-taking behavior, Social activities

## Abstract

**Background:**

Risk aversion due to depression is common among older adults, and social participation is associated with improved mental health and a lower risk of late-life depression. However, little is known about the connection between participation in social activities and risky financial decisions among adults with depression. Thus, we aim to examine the connection between participation in social activities and taking financial risks and investing in risky financial assets (with high-return potential) in such individuals, differentiated by age and gender. The study also focuses on analyzing the percentage of investors within each social activity, their attendance frequency, and motivation.

**Methods:**

The data was obtained from the Survey of Health, Ageing and Retirement in Europe (SHARE) database Wave 2 (2006–2010). The study included 8,769 individuals aged 50 + with depression caseness, from 15 European countries and Israel who answered the question on participation in social activities and reported financial risk-taking intentions or behaviors (investing in stocks or shares, mutual funds or managed investment accounts, and both). The study utilized Pearson chi-square, odds ratios, Z, and hierarchical logistic regression tests.

**Results:**

The odds for taking financial risks and investing in risky financial assets were higher for those participating in social activities compared to those who did not, on both intentional (by 173%) and behavioral (by 240–397%) levels. Such social activities (attended at least once a week, without financial motivation) have been shown to be primarily represented by educational or training courses — where 33% of participants invested in risky financial assets. The connection persisted after controlling for gender, age, marital status, children, income.

**Conclusions:**

By overcoming the subjects’ financial risk aversion, participation in social activities may help improve mental health in individuals aged 50 + with depression caseness. This has important implications for policymakers in healthcare, who by updating healthcare policies can fund and facilitate participation in social activities. As a result, the national healthcare system may benefit from lower hospitalization-related expenses, and generate higher cash flows into the country’s economy using the population’s renewed interest in investing available funds. These results are relevant in the wake of COVID-19 that increased loneliness and depression rates.

**Supplementary Information:**

The online version contains supplementary material available at 10.1186/s13584-024-00621-z.

## Background

Major depression is a prevalent and severe mental illness that negatively affects an individual's cognitive function and behaviors [1–3] and is expected to become the largest contributor to the disease burden by 2030 [4]. In recent years, depressive symptoms have increased among the general population due to the coronavirus disease 2019 (COVID-19) outbreak [5], which emerged as a global pandemic and became an international public health major concern [6]. Older individuals who were among the most at-risk populations [7] had twice the odds of depressive symptoms during the pandemic compared with the pre-pandemic period due to loneliness and other COVID-19 stress-related factors [8]. The pandemic forced millions of people worldwide to alter their lifestyles and social participation.

Social participation, defined as involvement in various social activities [9], is beneficial to the psychological health of older adults (65 +) and is associated with a lower risk of late-life depressive symptoms [10]. Previous studies have shown that active participation of individuals aged 60 + in religious activities, political groups, clubs, and volunteering is a predictor of better self-rated health and life satisfaction, as well as decreased depression and mortality [11–16]. Participation in social activities and mental/physical health are positively correlated [14, 17].

Depressed individuals have a more negative view of reality [18], which could impact their economic behavior. For example, previous studies found associations between depression and decreased sensitivity to reward, which appear to underlie a failure to maximize potential monetary earnings [19, 20]. Non-depressed individuals changed their response patterns during the reward condition (verbal recognition task under three monetary payoff conditions: neutral, reward, and punishment) to maximize their earnings, whereas depressed subjects did not [19].

Related to this decreased responsiveness to rewarding stimuli are risk preferences expressed by depressed subjects. Risk preferences are understood here as preferences for actions or behaviors that, being rewarding, involve a possibility of loss. Such preferences may also have economic implications: individuals may display the tendency to engage in activities that have a higher variance in returns, no matter if such activities involve gains or losses [21]. When studying the subjects’ risk preferences, we should certainly differentiate between their revealed and stated risk preferences. While revealed risk preferences are observed in the subjects’ actual behavior, their stated risk preferences are revealed through self-reports. As is commonly the case with self-reported measures, they may lack objectivity and generalizability, and would surely benefit from empirical evidence. To illustrate, a study into how depressive episodes are associated with risk preferences and risk-taking behaviors reveals an association between depression and stated risk preferences, while displaying no correlation with revealed (or behavioral) risk preferences [22].

However, the present study, like most other psychological and economic studies of risk preferences, analyzes the subjects’ financial risk-taking based on their self-reports. Other scholars, using the same sort of data, investigate age-related changes in risk attitudes [23], correlations between financial and health risk-taking behaviors (such as participating in active sports) [24], reasons for individual differences in risk preferences (in the sample of 11,000 twins) and their correlations with observable choices and outcomes [25].

Stated risk preferences in depressed subjects may be shaped by their fear of failing that causes them to avoid taking risks in their professional lives or personal relationships [26]. Depressed individuals have unrealistic future expectations and are risk-averse [18, 27]. This behavioral change affects their decisions, which may lead to reduced investment in risky financial assets and could be translated into decreased willingness to acquire stocks or shares with potentially high returns [28, 29]. Depression per se is an obstacle to managing everyday tasks, including economic decision-making and specifically investing in risky financial assets.

Although studies have shown the connection between depression and risk-averse financial behavior, and that social participation buffers the negative effects of depression, the link between participation in social activities of adults with depression and their risky financial decisions has yet not been explored. Exploring this link is crucial since it could affect older individuals’ well-being, given the rapid increase in depression incidences among this population following the COVID-19 outbreak.

For Israel, the well-being of older individuals is of particular importance. The Israeli elderly population has been growing at twice the rate of the overall population — which has implications for potential healthcare expenses [30]. In Israel, public household expenditures per person on health are the highest in the 50 to 80 + age groups compared to children and teenagers. This constitutes the highest financial load on health insurers (or health maintenance organizations, HMOs) [31]. Preserving the health of these age groups would relieve the burden on healthcare providers and reroute the costs available to them to other channels.

In their turn, these financial decisions made by older individuals are important to the Israeli economy. This is because in 2010 individuals aged 55 to 64 where the highest income earners in Israel, with their share in the top decile based on per capita income being 23.4%. On a broader scale, the second and third highest numbers of those in the top decile per capita income were found in adjacent age groups, 45–54 (15.1%) and 65 + (15%) [32]. In 2022 however, the income distribution across different age groups in Israel was slightly different, with the highest share in the top decile being registered for the 40 to 50 age group. At the same time, the general tendency has remained unchanged — the probability of belonging to the higher wage percentiles increases with age and remains high compared to younger age groups [33].

Thus, the primary objective of this paper was to examine whether individuals aged 50 + with depression caseness who participate in social activities invest differently in risky financial assets compared to individuals who do not participate in social activities (by gender within age groups). We do not view these (possible) connections as causal – rather as informative in characterizing individuals aged 50 + with depression caseness. Our secondary goals were: 1. To examine the financial risk-taking intentions among individuals participating compared to those not participating in social activities (by gender within age groups). 2. To map the percentage of investors in risky financial assets among the participants of each social activity. 3. To analyze how often (frequency) the investors attend each social activity and what motivates them to participate. Pursuing the goals above could provide healthcare authorities with a rationale to update their policies and encourage individuals aged 50 + to engage or increase their participation in social activities. This could improve their financial future and well-being, especially in the wake of the COVID-19 pandemic that has increased loneliness and depression rates.

## Methods

### Data source: SHARE survey

Data from the Survey of Health, Ageing and Retirement in Europe (SHARE) Wave 2 [34–36] were analyzed (Additional file 1, Sect. 1: SHARE questions used for the paper). Wave 2 was conducted between 2006–2010 and contains information regarding depression, social activities, risky financial assets, and risk aversion. Since it is the first wave that contains information regarding risk aversion, it was chosen for this study. Data were collected for 15 countries: Austria, Belgium, Czech Republic, Denmark, France, Germany, Greece, Ireland, Israel, Italy, Netherlands, Poland, Spain, Sweden, and Switzerland. The next wave with a section on risk aversion was Wave 4 (2011–2014). Even though the questionnaire employed in Wave 4 longitudinal design was the same as in Wave 2, there were differences in their scale of measurement that could render them incomparable. First, the activities questioned in Wave 2 encompasses a one-month period (activities engaged in over the last month), while in Wave 4 — a twelve-month range (over the last twelve months). In addition to different ranges, the two waves differ in the nature of activities listed as answer options to choose from. While all the activities offered in Wave 2 question are social, Wave 4 options include activities that do not presuppose social interaction. Other activities from Wave 2 are not included in the answer options in Wave 4. Finally, the countries conducting baseline interviews in SHARE Waves 2 and 4 are not the same. Most importantly, the list of countries in Wave 4 does not include Israel and would thus not provide a basis for comparison [37, 38].

SHARE [39] is an international consortium and longitudinal database that contains data on mental and physical health, social networks and activities, socioeconomic status, and well-being of about 140,000 individuals aged 50 or older (around 530,000 interviews). It was established in 2002. SHARE covers 28 European countries and Israel.

Although SHARE is a longitudinal survey, this study focuses on only one of its cycles (Wave 2, 2006–2010). The reason for selecting no more than one wave is rotted in the average holding period of risky financial assets. While in 1976 the holding period of shares was reported to be 3.9 years (world average), it shrank to less than a year by 2005, and to a mere 7.4 months by 2015 [40]. A similar pattern can be observed in the holding period of another type of risky financial asset, such as stocks: under a year since the 2000s, and down to 9.6 months by 2018 [41, 42]. In view of risky financial assets being held for an average of under a year, one five-year wave is a sufficient period to explore financial risk-taking behaviors.

### EURO-D depression scale

The EURO-D scale [43] was originally developed with the aim of creating a unified depression symptoms scale derived from various instruments used to assess late-life depression across different European countries. The scale includes 12 common depressive symptoms: feeling sad, pessimism, suicidality, guilt, trouble sleeping, loss of interest, irritability, loss of appetite, fatigue, difficulty in concentrating, loss of enjoyment, and tearfulness. SHARE administers the EURO-D scale to its respondents, and seeks to elicit ‘case of depression’, i.e. depression caseness, based on the presence of ≥ 4 of the symptoms (Additional file 1, Subsection 1.1: EURO-D questions). According to the approach adopted by SHARE, the scale score below 4 is categorized as ‘not depressed’ [44]. To be consistent with SHARE methodology, the present study categorizes individuals as having depression caseness if they have at least four depressive symptoms. Based on this approach, 25% of the Wave 2 sample have depression caseness — a result that aligns with the data reported by the World Health Organization (WHO) [45]. The same cutoff point of four or more depressive symptoms has been considered optimal and adopted in other studies investigating late-life depression [46–49]. These previous studies have also testified to cross-cultural validity of the EURO-D scale and demonstrated its high internal consistency [43, 46–49].

To ensure the robustness of the cutoff method of ≥ 4 EURO-D scale symptoms, we conducted additional analyses of dependence and connection between participating in social activities and financial risk-taking intentions and behaviors for individuals with cutoff points of ≥ 3 and ≥ 5 depressive symptoms. The results revealed no significant differences in the connection between participation in social activities and risk preferences in individuals with ≥ 3, ≥ 4 and ≥ 5 depressive symptoms (95% confidence interval (95% CI) highly overlapping) (Additional file 1, Sect. 2: Dependence and connection between participating in social activities and financial risk-taking intentions and behaviors in individuals with ≥ 3, ≥ 4, and ≥ 5 depressive symptoms). Therefore, the Results section below will only discuss the analysis conducted with cutoff point of four depressive symptoms or more.

### Social activities

The SHARE Wave 2 questionnaire targeted participation in seven activities during the previous month, such as voluntary or charity work, caring for a sick or disabled adult, providing help to family, friends or neighbors, attending an educational or training course, going to sport, social or other kind of club, and taking part in activities of a religious organization (church, synagogue, mosque etc.) etc. In addition, the questionnaire measured the frequency of attending these social activities, such as “*Almost daily*”, “*Almost every week*” or “*Less often*”, and the motivation for the social involvement, such as “*To meet other people*”, “*To contribute something useful*”, “*Because I am needed*”, “*To earn money*” or “*To use my skills or to keep fit*” (Additional file 1, Subsection 1.2: Social activities questions).

Based on our analysis, among individuals with depression caseness in the Wave 2 SHARE sample used in the present study, 37% reported participating in at least one social activity. Participation in at least two social activities was reported by a mere 13% of study subjects. As for the majority of the participants (63%), they opted for the ‘None of the above’ answer thus indicating they did not take part in any social activity. Considering a relatively limited sample size, it seems productive to investigate the connection between participation in social activities and risk preferences in individuals reporting participation in at least one social activity. This approach is also in line with the findings that participation in at least one social activity (compared to two or more activities) is common among Europeans aged 50 and above [50].

To ensure the robustness of the results, we additionally analyzed the dependence and connection between participating in social activities and risk preferences in individuals among the participants with depression caseness who reported participation in at least two activities (Additional file 1, Sect. 3: Dependence and connection between participating in social activities and financial risk-taking intentions and behavior in individuals participating in at least one / at least two social activities).

### Investments in risky financial assets

Individuals were asked a question regarding their savings or investments possessions: “*Which, if any, of these savings and investments do you [or your/ husband/wife/partner] have?*” Investment in risky financial assets was defined as stocks held directly or stocks held through mutual funds and investment accounts [51, 52]. Accordingly, two questions were considered in this study: 1. “*Do you [or your partner] currently have any money in stocks or shares (listed or unlisted on stock market)?*”, 2. “*Do you [or your partner] currently have any money in mutual funds or managed investment accounts?*” with a “*Yes*” or “*No*” as answer (Additional file 1, Subsection 1.3: Risky financial assets questions). Risky financial assets investments were examined in three groups: 1. stocks or shares, 2. mutual funds or managed investment accounts, and 3. both.

Given that the questions regarding risky financial assets encompassed assets held by the respondent’s partner as well, it seemed important to ensure that the preferences and influence of the spouse are negligible in driving the results. This called for analyzing the relationship between participation in social activities and financial risk-taking behavior in respondents who report having no partner, in addition to the analysis of this relationship in participants of all marital status. For this we assigned participants selecting one of the answer options below: separated from a spouse, never married, divorced, or widowed. However, the analysis yielded no significant differences in the results with and without the limitation. The results for the connection between participating in social activities and financial risk-taking behavior in participants reporting no partner (separated, never married, divorced, or widowed) displayed a 95% CI overlap with the results from the participants of all marital status (Additional file 1, Sect. 4: Dependence and connection between participating in social activities and financial risk-taking behavior for participants of all marital status, and participants reporting no partner). This suggests a negligible influence of marital status on the dependence and connection between the two variables under study. Additionally, imposing a restriction on marital status drastically reduced the sample size, which would limit the possibility of stratification by gender within age groups. For these reasons, the Results section below will only discuss the analysis conducted on the entire study population, without the restriction on marital status.

### Risk-taking intentions and risk aversion

In the SHARE Wave 2 questionnaire, participants were asked questions regarding their risk-taking intentions i.e., “*When people invest their savings, they can choose between assets that give low return with little risk to lose money, for instance, a bank account or a safe bond, or assets with a high return but also a higher risk of losing, for instance, stocks and shares. Which of the statements on the card comes closest to the amount of financial risk that you are willing to take when you save or make investments?*” (Additional file 1, Subsection 1.4: Risk aversion question). Individuals were considered willing to take financial risks when they replied that they were willing to take substantial, above average, or average financial risk (expecting to earn substantial, above average, or average returns). Individuals were considered risk averse when they replied, “*Not willing to take any financial risks*”.

Potential connection between financial risk-taking intentions and behaviors, and participation in social activities is viewed in this paper as having a descriptive rather than causal nature.

### Sample characteristics and sampling process

Wave 2 of the SHARE dataset comprises responses from 37,143 individuals. Being a study of health, ageing and retirement in Europe, the SHARE database contains data for individuals who are 50 years or older at the time of sampling [53]. However, its Wave 2 dataset includes responses from individuals aged 14 to 102. At stage 1, we limited the sample to those who (i) disclosed their age at the time of the interview, and (ii) answered the EURO-D scale question (total number 35,983). At stage 2, we further limited the sample to the respondents aged 50 and above (total number 34,958). Finally, we limited the study subjects to those of them who were categorized by SHARE as having depression caseness based on the EURO-D depression scale (the presence of ≥ 4 of the symptoms, as explained in the Methods above). This final study sample included 8,769 individuals whose responses were used in the present study.

8,769 individuals aged 50 and older with depression caseness who did or did not participate in at least one social activity were included in this study. The average age of participants was 66.8 years (standard deviation = 10.87). These individuals were stratified by gender within four age groups: 50–59, 60–69, 70–79, and 80 + (Table 1).

**Table 1:** Basic demographics of participants, age and gender

Characteristic	N (% gender; % age group)		
**Age group**	**Male**	**Female**	**Both genders**
50–59	803 (28.7)	1,997 (71.3)	2,800 (31.9)
60–69	755 (30.5)	1,719 (69.5)	2,474 (28.2)
70–79	731 (33.3)	1,461 (66.7)	2,192 (25.0)
80 +	456 (35.0)	846 (65.0)	1,302 (14.9)
Total	2,746 (31.3)	6,023 (68.7)	8,769 (100.0)

It should be noted that the terms used in Table 1 are inherited from the SHARE database. While referring to gender, the SHARE database distinguishes between males and females — instead of men and women. We recognize that gender identity is constructed socially (not biologically) and is not confined to a binary opposition [54]. However, we find it important to align our analysis with the original wording used in the SHARE database. For this reason, the current paper will refer to gender in the same way this variable is categorized in the SHARE database.

Individuals categorized with depression caseness that did (3,214) or did not (5,396) participate in at least one social activity were analyzed for their financial risk-taking intentions (willing or not willing to take financial risks) and behaviors (invested or not invested in risky financial assets). At the next stage, the study identified the percentage of investors among those participating in each social activity. In addition, the frequency (almost daily, weekly, or less often) and the motivation for participating in social activities were analyzed (Table 2). As described, only individuals with depression caseness (determined by the EURO-D Scale) were analyzed.

**Table 2:** Study parameters, answer options & distribution of responses

Study parameters	N (%)
Social activities:	
Not participate	5,396 (62.7)
Participate (at least in one social activity)	3,214 (37.3)
Activity types:	
Done voluntary or charity work	701 (13.8)
Cared for a sick or disabled adult	683 (13.4)
Provided help to family, friends or neighbors	1,149 (22.6)
Attended educational or training course	430 (8.5)
Gone to sport, social or other kind of club	1,023 (20.1)
Taken part in activities of a religious organization (church, synagogue, mosque etc.)	897 (17.7)
Taken part in a political or community-related organization	200 (3.9)
Risk-taking intentions:	
Take substantial financial risks	50 (0.8)
Take above average financial risks	155 (2.6)
Take average financial risks	741 (12.3)
Not willing to take any financial risks	5,100 (84.3)
Financial assets:	
Without	5,325 (87.3)
With	774 (12.7)
Asset types:	
Stocks or shares	534 (57.1)
Mutual funds or managed investment accounts	402 (42.9)

The female-to-male ratio in the study sample was approximately two to one (68.7% and 31.3%, respectively). It is well-established that females are more inclined to participate in surveys compared to males [55, 56]. Additionally, the prevalence of major depression among females is nearly double that of males [57, 58]. It is worth noting that throughout each analysis conducted in this study, the gender ratio was examined and consistently found to align with that of the general population.

In the present study, one of the objectives was to determine the percentage of investors in risky financial assets among the participants of each social activity. For this, it was important to calculate the number of study subjects who held financial assets split into two types — (i) stocks or shares, and (ii) mutual funds or managed investment accounts. For the purposes of this study, we excluded from the calculations the participants who responded to both questions on financial assets with “*Don’t know*” or “*Refusal*” or left them unanswered. The rest of the participants were considered as those who answered the asset questions (positively or negatively). From these, we subtracted the number of participants with negative answers to both questions on financial assets — which left us with the number of participants who reported holding financial asset(s). The same approach was used when calculating the frequency of attending social activities, and the motivation for involvement among investors in risky financial assets.

Although our main population of interest is represented by individuals with depression caseness, we also analyzed the dependence and connection between participating in social activities and financial risk-taking intentions and behaviors in two samples: (i) the entire sample including those with and without depression caseness, and (ii) individuals without depression caseness (Additional file 1, Sect. 5: Dependence and connection between participating in social activities and financial risk-taking intentions and behavior among individuals with and without depression caseness, without depression caseness, and with depression caseness). However, there were no significant differences in the results obtained for the three groups (95% CI highly overlapping). Therefore, the Results section below will only discuss the dependence and connection between the variables of interest registered for the individuals with depression caseness. The implications for these findings will be discussed in the Discussion section.

### Statistical analysis

The analysis was carried out using SPSS Version 25.0 (IBM Corp., Armonk, NY, USA). Pearson chi-square (*χ*^2^(degrees of freedom)) and odds ratio (OR) (with 95% CI) statistical methods were conducted to examine the dependence and connection between social activities and financial risk-taking intentions/behavior among individuals with depression caseness and when stratifying by gender within age groups. In cases where the expected count was less than five, Fisher's exact test was performed (instead of Pearson chi-square). *p* values < 0.05 were considered significant.

Z-test with Bonferroni Correction *p* < 0.0024 was conducted for comparing independent proportions (expressed as percentages) of investors in risky financial assets within social activities.

In addition, it was decided to check for control variables that could contribute to shaping the connection between participating in social activities and risk preferences in individuals aged 50 and above with depression caseness. For this, we performed a hierarchical binary logistic regression analysis. The control variables included in the analysis were those of age, gender, marital status, number / presence of children and average household monthly income (Euro (EUR)/1000) (Additional file 1, Subsections 1.5, 1.6 and 1.7). This analysis (and this study in general) did not seek to establish cause-and-effect relationships between the variables.

## Results

### Dependence and connection between social activities and financial risk-taking intentions

**Fig. 1:**
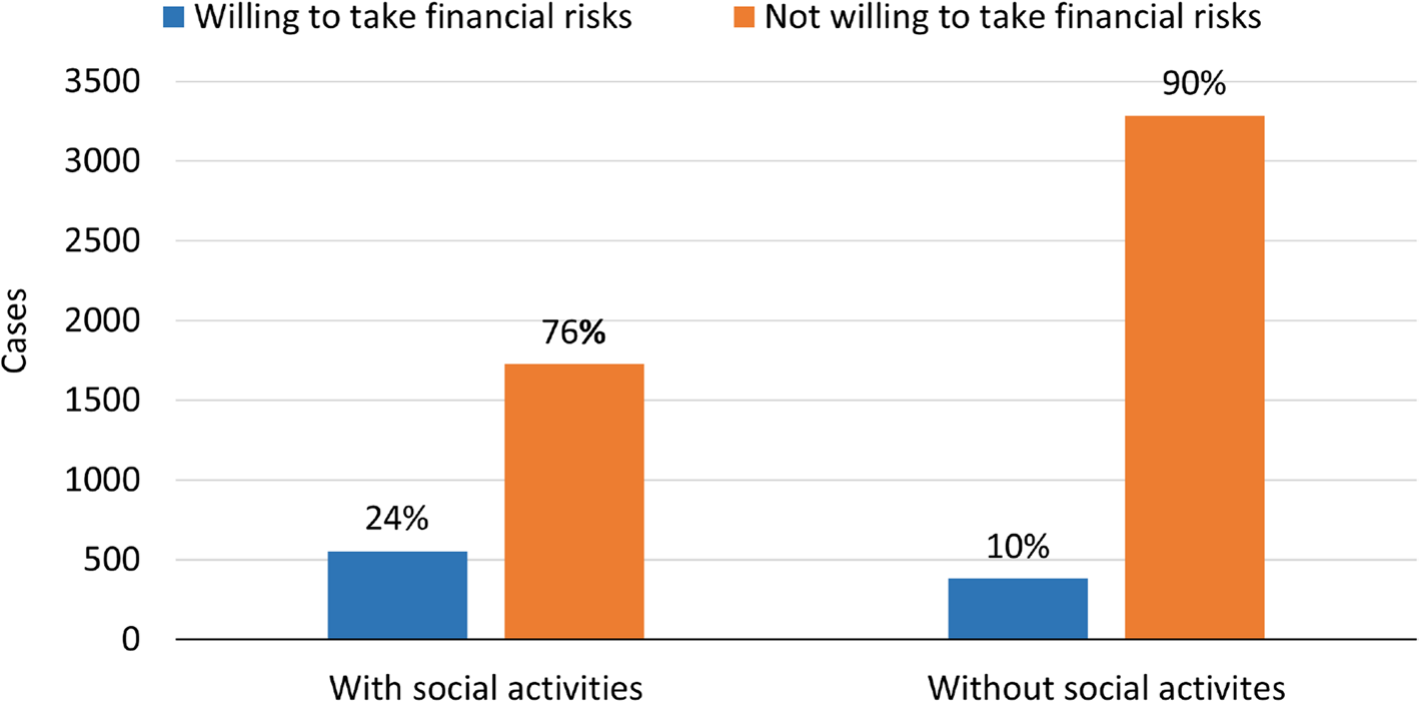
Financial risk-taking intentions as a function of participating in social activities

To explore the link between participation in social activities and financial risk-taking intentions among individuals with depression caseness, we examined individuals who were willing or not willing to take financial risks (Fig. 1).

Chi-square analysis revealed that there was significant dependence and connection between participation in social activities and financial risk-taking intentions among individuals with depression caseness. The odds for financial risk-taking intentions were 173% higher among individuals participating in social activities, as compared to individuals who did not participate in social activities (*χ*^2^(1) = 200.13, *p* < 0.001, OR = 2.73, 95% CI: 2.37–3.15).

At the next stage, the study subjects who reported participating in social activities and having financial risk-taking intentions were stratified by age and gender. This stratification demonstrated significant dependence and connection between participation in social activities and financial risk-taking intentions for both genders across all age groups (OR ranged from 1.77, 95% CI: 1.06–2.96, *p* = 0.030 to 3.47, 95% CI: 2.39–5.03, *p* < 0.001. Chi-square ranged from *χ*^2^(1) = 4.33, *p* = 0.037 to *χ*^2^(1) = 44.87, *p* < 0.001). Additionally, since the 95% CI highly overlapped, there were no significant differences in the results obtained for both genders and different age groups. (Additional file 1, Subsection 6.1: Stratification by gender within age groups: Dependence and connection between participating in social activities and financial risk-taking intentions). These findings suggest that individuals aged 50 and above participating in social activities are more willing to take financial risks compared to those who do not participate in social activities, with no exceptions in terms of age groups and gender.

To control for other factors that could influence the connection between participation in social activities and financial risk-taking intentions, we considered such control variables as gender, age, marital status, number of children and average household monthly income. Of these, significant predictors for the connection under study were the gender, age, number of children, and the average household monthly income (EUR/1000). For males, the odds for being willing to take financial risks were 58% higher compared to females (OR = 1.58, *p* < 0.001). Age-wise, each 1-year increase in age lowered the odds of being willing to take financial risks by 4% (OR = 0.96, *p* < 0.001). Similarly, the odds of an individual being willing to take financial risks were found to decrease as (s)he had more children – by 14% per child (OR = 0.86, *p* < 0.001). In terms of income, it also displayed a significant connection with financial risk-taking intentions: each 1-thousand euro increase in the average monthly household income increased the odds of taking financial risks by 0.3% (OR = 1.003, *p* < 0.001). After controlling for the variables above, the odds for being willing to take financial risks were 140% higher among individuals participating in social activities compared to those who did not participate in these activities (OR = 2.40, *p* < 0.001). One control variable – marital status – was found not to be a significant predictor for the connection under study (Additional file 1, Subsection 7.1: Social activities and financial risk-taking intentions (with control variables)). For all control variables, no significant differences were registered in the connection between participating in social activities and financial risk-taking intentions with and without them (95% CI highly overlapped).

### Dependence and connection between social activities and financial risk-taking behavior

**Fig. 2:**
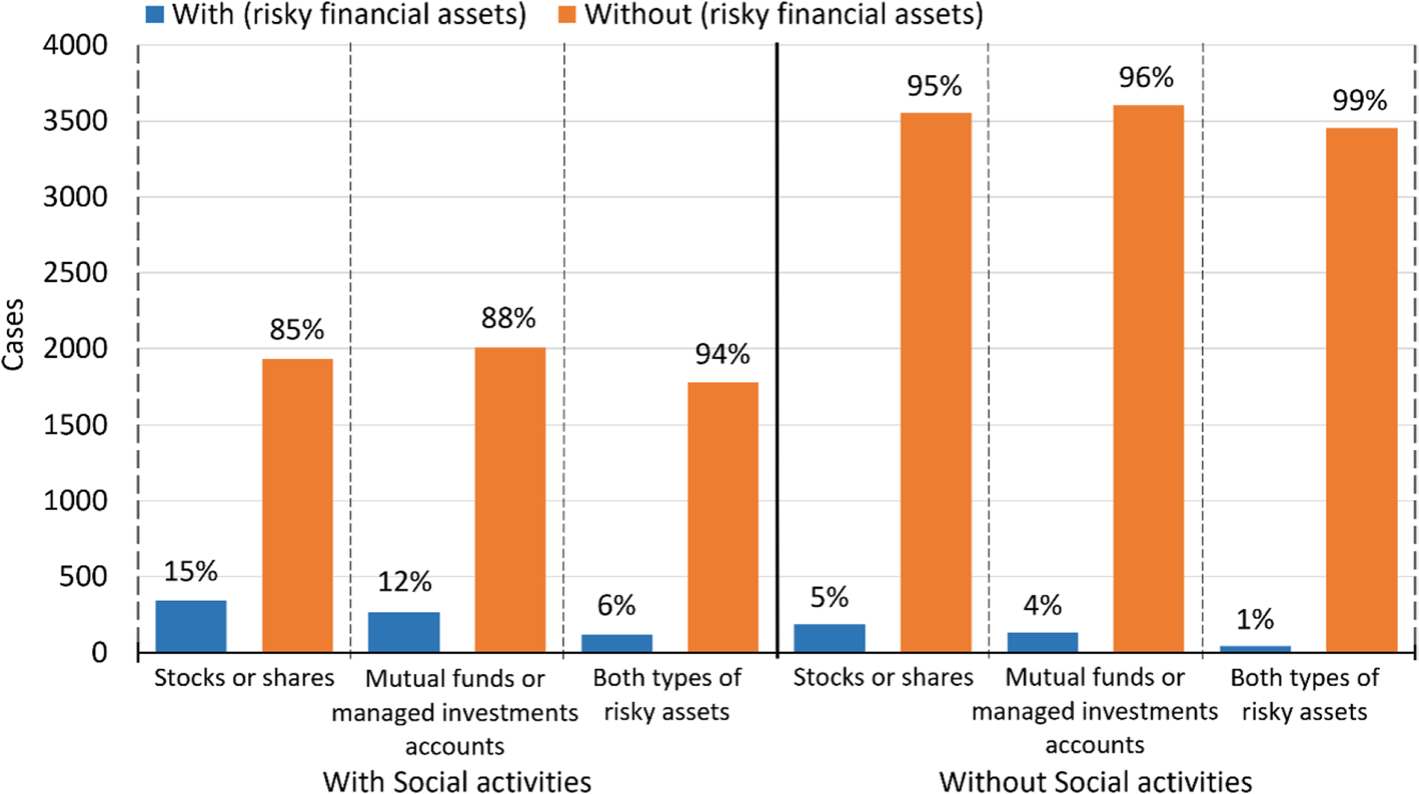
Financial risk-taking behavior as a function of participating in social activities

In addition to analyzing financial risk-taking intentions, we examined how participating in social activities connects to financial risk-taking behavior as a form of investing in risky financial assets. For this purpose, individuals with depression caseness with and without social activities were examined for their investments in stocks or shares, mutual funds or managed investments accounts and both types of assets (Fig. 2).

There was significant dependence and connection between social activities and investing in risky financial assets among individuals with depression caseness. The odds for individuals investing in stocks or shares were 240% higher (*χ*^2^(1) = 180.32, *p* < 0.001, OR = 3.40, 95% CI: 2.82–4.10), the odds for individuals investing in mutual funds or managed investments accounts were 260% higher (*χ*^2^(1) = 151.12, *p* < 0.001, OR = 3.60, 95% CI: 2.90–4.47) and the odds for individuals investing in both types of risky assets were 397% higher (*χ*^2^(1) = 97.97, *p* < 0.001, OR = 4.97, 95% CI: 3.50–7.05) among individuals participating in social activities compared to those who did not.

At the next stage, the study subjects who reported participating in social activities and engaging in financial risk-taking behaviors were stratified by age and gender. This stratification demonstrated significant dependence and connection between participation in social activities and engagement in financial risk-taking behaviors for both genders in such age groups as 50–59, 60–69 and 70–79 (OR ranged from 1.89, 95% CI: 1.01–3.54, *p* = 0.048 to 11.68, 95% CI: 3.30–41.43, *p* < 0.001. Chi-square ranged from *χ*^2^(1) = 4.01, *p* = 0.045 to *χ*^2^(1) = 42.38, *p* < 0.001). As for the age group 80 + , statistical analysis did not reveal significant dependence and connection for males investing in mutual funds or managed investments accounts, and for females investing in stocks or shares. Additionally, no significant dependence and connection was registered between participating in social activities and investing in both types of risky financial assets among both males and females in this age group. At the same time, the 80 + age group revealed significant dependence and connection between participating in social activities and investing in (i) stocks or shares among males (OR = 2.82, 95% CI: 1.09–7.33, *p* = 0.033, Fisher's exact test *p* = 0.041), and (ii) mutual funds or managed investments accounts among females (OR = 5.93, 95% CI: 2.60–13.51, *χ*^2^(1) = 22.44, *p* < 0.001). Throughout all age groups, the analysis revealed no significant differences in the results obtained for males and females (95% CI highly overlapped) (Additional file 1, Subsection 6.2: Stratification by gender within age groups: Dependence and connection between participating in social activities and financial risk-taking behavior). These findings suggest that in all age groups under analysis and in both genders, individuals participating in social activities are more willing to invest in risky financial assets compared to those who do not participate in social activities.

To control for other factors that could influence the connection between participating in social activities and investing in *stocks or shares*, we considered such control variables as gender, age, marital status, number of children and average household monthly income. Of these, significant predictors for the connection under study were the gender, age and the average household monthly income (EUR/1000). For males, the odds for investing in stocks or shares were 42% higher compared to females (OR = 1.42, *p* = 0.029). Age-wise, each 1-year increase in age lowered the odds of investing in stocks or shares by 2% (OR = 0.98, *p* = 0.003). In terms of income, it also displayed a significant connection with financial risk-taking behavior: each 1-thousand euro increase in the average monthly household income increased the odds of investing in stocks or shares by 0.3% (OR = 1.003, *p* < 0.001). After controlling for the variables above, the odds for investing in stocks or shares were 307% higher among individuals participating in social activities compared to those who did not participate in these activities (OR = 4.07, *p* < 0.001). Two control variables – marital status and number of children – were found not to be significant predictors for the connection under study (Additional file 1, Subsection 7.2: Social activities and investments in stocks or shares (with control variables)).

In addition to stocks and shares, this study also considered another type of risky financial assets – *mutual funds and managed investments accounts*. For this reason, it appeared crucial to control for factors that could influence the connection between participating in social activities and investing in this type of assets. The control variables included in the analysis were those of gender, age, marital status, number of children and average household monthly income. Of these, significant predictors for the connection under study were the age and marital status. Age-wise, each 1-year increase in age lowered the odds of investing in mutual funds or managed investments accounts by 2% (OR = 0.98, *p* = 0.024). As for participants with different marital status, those reporting being in a relationship had 53% higher odds for investing in mutual funds or managed investments accounts compared to respondents with no relationship (OR = 1.53, *p* = 0.022). After controlling for the variables above, the odds for investing in mutual funds or managed investments accounts were 282% higher among individuals participating in social activities compared to those who did not participate in these activities (OR = 3.82, *p* < 0.001). Three control variables – gender, number of children and average household monthly income – were found not to be significant predictors for the connection under study (Additional file 1, subsection 7.3: Social activities and investments in mutual funds or managed investments accounts (with control variables)).

To look into potential influences of control variables on the respondents’ financial risk-taking behaviors, we decided to analyze the role played by the controls on the connection between participating in social activities and investing in *at least one risky financial asset*. This analysis did not differentiate between types of assets, focusing instead on at least one positive answer to the question about investing in risky financial assets. The control variables included in the analysis were those of gender, age, marital status, presence of children and average household monthly income. Of these, significant predictors for the connection under study were the age and average household monthly income (EUR/1000). Age-wise, each 1-year increase in age lowered the odds of investing in at least one risky financial asset by 2% (OR = 0.98, *p* = 0.001). In terms of income, it also displayed a significant connection with financial risk-taking behavior: each 1-thousand euro increase in the average monthly household income increased the odds of investing in at least one risky financial asset by 0.3% (OR = 1.003, *p* < 0.001). After controlling for the variables above, the odds for investing in at least one risky financial asset were 292% higher among individuals participating in social activities compared to those who did not participate in these activities (OR = 3.92, *p* < 0.001). Three control variables – gender, marital status and presence of children – were found not to be significant predictors for the connection under study (Additional file 1, Subsection 7.4: Social activities and investments in at least one risky financial asset (with control variables)).

For all control variables, no significant differences were registered in the connection between participating in social activities and all risk-taking behaviors with and without these controls (95% CI highly overlapped).

### Percentage of investors in risky financial assets for each social activity

**Fig. 3:**
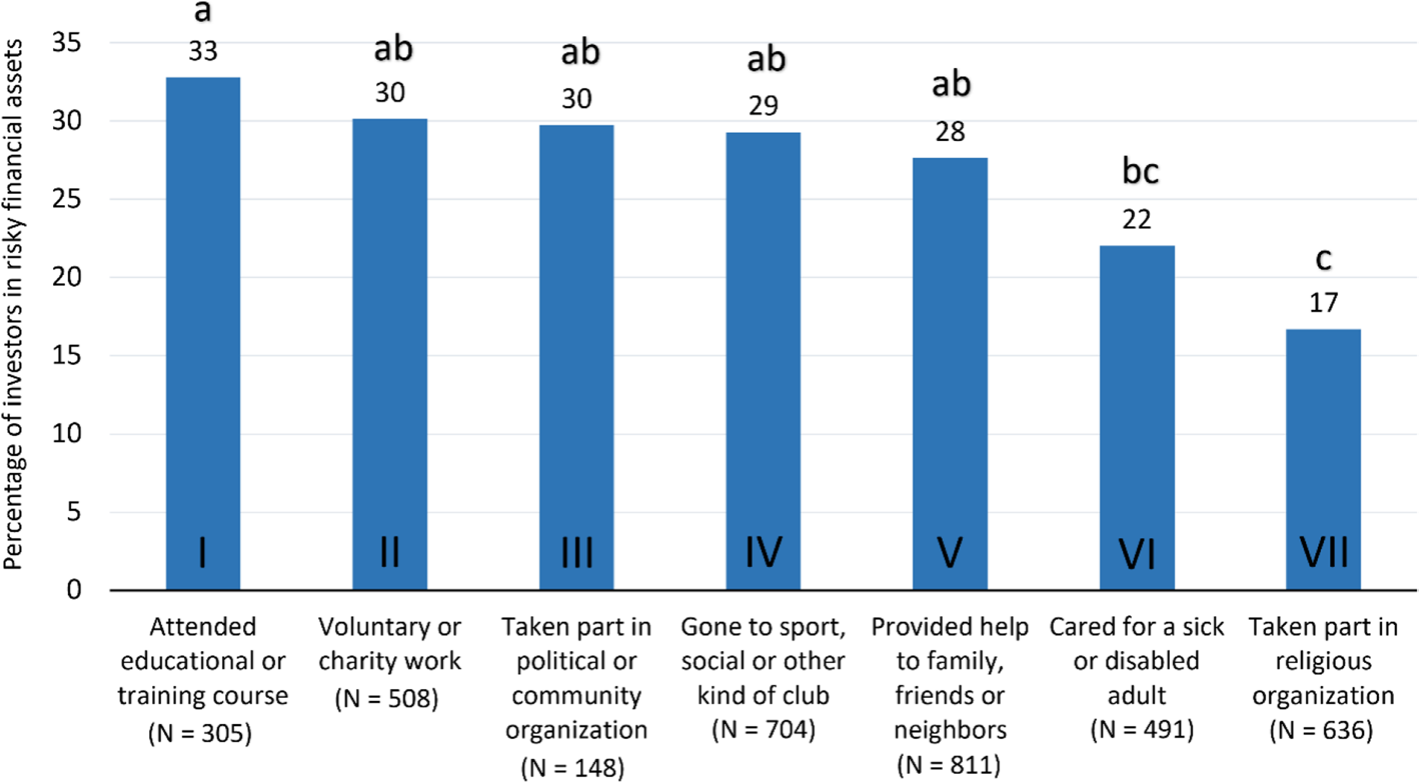
Percentage of investors in risky financial assets within social activities. The figure uses a compact letter display (CLD), with any treatments not sharing a letter being significantly different, *p* < 0.0024 (Bonferroni Correction)

In order to evaluate which social activities had the highest percentage of investors in risky financial assets, the number of cases of investors was analyzed relative to the total participants (investing or not investing in risky financial assets) within each social activity (Fig. 3).

The percentage of investors in risky financial assets in I was higher than and significantly different from VI and VII. The percentage of investors in risky financial assets in I to V was higher than and significantly different from VII.

### Frequency of attending social activities among investors in risky financial assets

To understand how often in the last four weeks individuals with depression caseness participated in each social activity, the frequencies for each social activity were analyzed (see Roman numerals as in Fig. 3).

Most individuals participating in social activities I, II, IV and VII attended “*Almost every week*” (50–67%). Most individuals participating in social activities III and V attended “*Less often*” (59% and 47%, respectively) and most individuals participating in social activity VI attended “*Almost daily*” (49%).

### Motivation for involvement in social activities among investors in risky financial assets

Among the investors who participated in social activities V and VI, 74% and 83%, respectively, attended the activities because they felt needed. For activities IV and I, 73% and 74%, respectively, attended in order to use their skills or to keep fit. For social activities VII, III and II, 41%, 68% and 84%, respectively, attended because they wanted “*To contribute something useful*”. In addition, 42% of the investors who participated in social activity VII attended “*To meet other people*”. The financial motivation was less common — only 1% to 4% of the investors who participated in social activities I, III, VI and VII attended in order “*To earn money*”.

### Dependence and connection between participation in least two social activities and financial risk preferences

As explained in the Methods above, it seems crucial to ensure the robustness of the results by additionally analyzing the dependence and connection between participating in social activities and risk preferences in individuals with depression caseness who reported participation in at least two activities. For this sample, significant dependence and connection was found between participation in social activities and *financial risk-taking intentions*. The odds for having financial risk-taking intentions were 287% higher for individuals participating in at least two social activities compared to those not participating in any social activities. Compared to individuals that participated in at least one social activity, these odds were also significantly higher (95% CI did not overlap) (Additional file 1, Sect. 3: Dependence and connection between participating in social activities and financial risk-taking intentions and behavior in individuals participating in at least one / at least two social activities).

As for *investing in risky financial assets*, it is represented by investing in (i) stocks or shares, (ii) mutual funds or managed investments accounts, and (iii) both types of risky assets. For participants in at least two social activities, significant dependence and connection was found between participation in social activities and investing in stocks or shares. The odds for individuals to invest in stocks or shares were 454% higher among individuals participating in at least two social activities compared to those who did not. These odds were also significantly higher than in individuals that participated in at least one social activity (95% CI did not overlap). A different finding was revealed for investing in mutual funds or managed investments accounts as well as both types of investments. Here, the analysis yielded no significant difference between the participants who engaged in at least one vs. at least two social activity/ies (with 95% CI overlapping). At the same time, we did register significant dependence and connection between these types of investments and participation in a social activity for the participants who engaged in at least one vs. zero social activity. The odds for the individuals in question to invest in mutual funds or managed investments accounts and in both types of risky assets were respectively 464% and 831% higher compared to individuals who did not participate in any social activity (Additional file 1, Sect. 3: Dependence and connection between participating in social activities and financial risk-taking intentions and behavior in individuals participating in at least one / at least two social activities).

## Discussion

This article examined the link between participating in social activities and investing in risky financial assets among individuals aged 50 + with depression caseness. Furthermore, it studied the differences in financial risk-taking intentions and mapped the percentage of investors within each social activity, their attendance frequency, and motivation for participation using the SHARE Wave 2 database. Previous studies showed that individuals with depression are averse to financial risk, have negative views about their future, and have difficulties managing their money [18, 59, 60]. Therefore, they invest less in risky financial assets (with potentially high returns) [28, 29]. This, in turn, may lower their pension wealth (in the presence of equity premium) and negatively affect their well-being [29, 61].

We found that individuals with depression caseness who participated in social activities were more willing to take financial risks, were less risk-averse, and invested more in risky financial assets compared to individuals who did not participate in social activities. Based on our findings, these connections between participation in social activities and financial risk preferences persisted after controlling for gender, age, marital status, number / presence of children and average household monthly income.

This finding is reinforced by the analysis we undertook for individuals with depression caseness who participated in at least two social activities. This category displayed an even higher willingness to take financial risks and lower risk aversion compared to individuals participating in at least one social activity. They also had significantly higher odds to invest in stocks or shares, compared to individuals participating in at least one social activity. These results indicate the robustness of our findings and suggest a clear connection between social interactions and financial risk preferences.

Another important result was yielded by the analysis of the dependence and connection between participation in social activities and financial risk preferences in three samples: (i) the entire population including those with and without depression caseness, (ii) individuals without depression caseness, and (iii) those with depression caseness. We observed a similar pattern in the results obtained for all three sample groups: participation in social activities was connected to higher risky financial preferences. These findings applied to both financial risk-taking intentions and behaviors, for samples with and without depression caseness. The effect size, as indicated by the odds ratio, was notably stronger in individuals with depression caseness for all risky financial preferences. However, since the 95% CI heavily overlapped between the groups, we found no significant differences in the results among them (Additional file 1, Sect. 5: Dependence and connection between participating in social activities and financial risk-taking intentions and behavior among individuals with and without depression caseness, without depression caseness, and with depression caseness). This raised the question of whether participation in social activities is connected to higher risky financial preferences regardless of depression caseness.

To examine this, we analyzed the percentage of financial risk preferences (both intentions and behaviors) between individuals not participating in social activities from the depression-caseness and non-depression-caseness samples. This served as a baseline unaffected by participating or not participating in social activities. Our findings revealed that among those who did not engage in social activities, individuals with depression caseness exhibited approximately half the level (in percentage) of financial risk-taking intentions (willingness to take financial risks) and behaviors (investing in stocks or shares, mutual funds or managed investments accounts and both types of assets), compared to individuals without depression caseness (Additional file 1, Sect. 8: Financial risk-taking intentions and behavior among individuals not participating in social activities, with or without depression caseness).

Thus, since individuals with depression caseness who did not participate in social activities had about half the positive risky financial preferences compared to those without depression caseness, joining social activities had a bigger impact on them. This may be because they tended to have lower reward motivation, thus being unwilling to engage in potentially rewarding behaviors. Such altered cost/benefit decision-making has been described in a study of effort-based decision-making in subjects with major depressive disorder and healthy controls [62]. In the current study, the introduction of an additional variable — participation in social activities — leveled the financial risk preferences in individuals with and without depression caseness, with the effect size for the former being even larger, probably due to a lower baseline. These results suggest the importance of participating in social activities, especially for individuals with depression caseness.

At the same time, it appeared crucial to control for factors that could influence the connection between the subjects’ participation in social activities and their financial risk preferences. In our paper, these factors included gender, age, marital status, presence/number of children and average household monthly income. One factor that played a significant role in shaping the subjects’ financial risk preferences was found to be gender. In males, the odds for being willing to take financial risks, and for investing in stocks or shares were higher compared to those of females (by 58% and 42%, respectively). These findings align with those reported in other studies on males being more risk seeking in making financial decisions than females [63–67]. Another significant factor was age. Each 1-year increase in age lowered the odds for being willing to take financial risks, and for investing in risky financial assets (by 4% and 2%, respectively). A similar trend was uncovered in a study of possible links between age and tolerance of financial risks. After analyzing over half a million risk questionnaires, its authors reported that risk tolerance slowly declined with age [68]. Finally, income has also been found to be a significant predictor for the connection under study. Each 1-thousand euro increase in the average monthly household income produced a slow rise in the odds of being willing to take financial risks and engaging in financial risk-taking behaviors (from 0.2% to 0.3%). Although the controls above were found to be significant predictors for the connection between the subjects’ participation in social activities and their financial risk preferences, no significant differences were registered in this connection with and without these controls.

As shown above, after controlling for these and other variables, the connection between participating in social activities and financial risk-taking intentions and behaviors still persisted in the SHARE individuals with depression caseness. This finding could be explained by a combination of two factors. On the one hand, studies demonstrate that individuals with depression have anhedonic characteristics, which lead to behavioral avoidance of potentially rewarding environmental contexts [60, 69], and also have pessimistic views and unrealistic expectations about their future [18]. These cause them to be more averse to financial risk [27] and to invest less in risky financial assets [28, 29]. On the other hand, since participation in social activities is associated with a lower risk of depressive symptoms in the elderly [70], participation in social activities serves to buffer the negative effects of their depression. Therefore, when an individual with depression caseness participates in social activities, social interactions may help alleviate some of the depressive symptoms and decrease pessimistic views regarding financial risk-taking intentions. The finding that individuals with depression caseness who did not participate in social activities invested less in risky financial assets could be explained by the claim that depressed individuals struggle to deal with the complexity of managing their money since depression impairs several cognitive abilities such as: attention-switching (takes more effort to refocus), working memory, planning, decision making, and causes overall lack of motivation [59]. The connection between social interaction and financial risk preferences was registered in both genders within all age groups studied (50 and above), indicating that participation in social activities among individuals aged 50 + with depression caseness can help overcome risk aversion, regardless of age or gender. At the same time, we recognize that financial risk preferences can be influenced by a lot of factors that are beyond this research (and beyond the range of data provided by the SHARE database). These factors may include the individual’s emotional state, cognitive capabilities and limitations, impulsivity, personality type and others.

Given that the elderly possess a significant share of the total population wealth (about 30%) [71, 72], and part of the depression symptoms is risk aversion, it is of interest to acknowledge that depression in this population may affect economic decisions and indirectly influence financial markets. Therefore, maintaining and enhancing social activity participation could assist in improving the well-being of older individuals [16, 73] and has the potential to encourage them to invest in risky financial assets. This, in turn, could route this population’s available funds to contribute to their country’s economy.

The percentage of investors in risky financial assets was the highest for those who “*Attended an educational or training course*” activity and was the lowest for those who have “*Taken part in activities of a religious organization (church, synagogue, mosque *etc*.)*” activity. Although participation in religious organizations may offer mental health benefits beyond those offered by other forms of social participation [14], individuals who consider themselves more religious are generally more risk-averse, which might explain the lowest percentage of investors in risky financial assets in this social activity type. This finding about religious individuals having a higher risk aversion is confirmed by other studies [74–76]. One possible explanation for the relation between religiosity and risk aversion is that it is mediated by anxiety [74]. A number of both classic and contemporary studies suggest that people may be motivated to become religious by the fear of uncertainty [77–79]. This role that uncertainty plays in religious beliefs can be attributed, among others, to an individuals’ attempt to distract themselves from frustrated goals, to restore their sense of control [79].

On the other hand, the highest percentage of investors in risky financial assets was registered among those who “*Attended an educational or training course*” activity. This could be explained by the positive impact that adults’ participation in educational programs has on their life satisfaction on the emotional, physical and cognitive/intellectual levels. When analyzing educational programs for older adults, a 2017 study specifically focused on physical activity programs, art programs and technologies learning programs. Having conducted semi-structured interviews, its author reported an increase in the participants’ evaluation of their social connectedness, optimistic outlook and gratitude. They attributed a higher emotional, physical, cognitive satisfaction to the possibility to interact with their peers or with younger people as part of an educational program [80]. Similar personal, mental, social and physical benefits were reported in another study into the role of lifelong education [81].

Of particular value for adults can be educational programs in information and communication technologies (ICT). Research shows that older adults who learned ICT and practically utilized their knowledge showed better self-control, and lower rates of depression and loneliness [82]. Better self-control seems a necessary prerequisite for the ability to take risks, including financial risks.

As seen from the analysis of the subjects’ motives for participation, in activities attended by a relatively high percentage of investors in risky financial assets, the investors were not motivated by the desire “*To earn money*”. Thus, although individuals invested more in risky financial assets and participated in these activities, their motivation for participation was not for profit. This demonstrates again that participation in social activities promotes investments in risky financial assets through mental and psychological paths [10, 16], which indirectly affect financial risk-taking intentions and behaviors.

As for risk-taking behaviors, they may be represented not just by owning risky financial assets (as considered in this paper), but also having supplementary health insurance (as approached by Cobb-Clark et al., [22]). According to their study on depression, risk preferences, and risk-taking behavior, depressed individuals often exhibit risk aversion in different behaviors related to financial risk-taking. Among these, the study considers such behaviors as having supplementary health insurance and owning risky assets. The absence of supplementary health insurance coverage may leave individuals vulnerable to unforeseen health-related expenses. On the other hand, deciding not to invest in risky financial assets, they may miss out on opportunities for wealth accumulation and financial growth. Moreover, this tendency towards financial risk aversion may compound existing financial insecurities (ibid.).

Therefore, interventions aimed at reducing financial risk aversion may have the potential to foster greater financial security and stability in the 50 + adult population. It should be borne in mind that the elderly have been historically considered a vulnerable population, both physically and economically. While life expectancy and quality of life have considerably improved within the lifetime of the Baby Boom generation, the income of many of them is still close to the poverty baseline [83]. A regulatory system providing them with adequate social support would thus improve the welfare of this age group [84]. Our findings have important implications for healthcare policy. Without suggesting causal relationships, they shed light on the link between participating in social activities and financial risk preferences and prioritize policy efforts. One way to do it is making the responsibility of national healthcare authorities to develop policies for facilitating the participation of adult populations in social activities most effective in reducing the participants’ financial risk aversion. These activities are primarily educational and training events. It stands to reason that the costs required for implementing the policies above and organizing these social events should be also covered by health organizations. After all, these events may result in improvements in the population's mental health, lower risk of late-life depression, and lower hospitalization rates — all of which will bring financial benefits to the national healthcare system. A secondary outcome generated by the social engagement policies above could be additional cash flows into the country’s economy. Improved mental health will help individuals to reduce their financial risk aversion and express more interest in channeling the funds available to them into the country’s economy.

This study had some limitations. Since the period of the risky financial assets acquisition was not measured, the claim that at least some of the population had risky financial assets before they participated in social activities cannot be rejected. However, this does not conflict with the finding that individuals participating in social activities invested more in risky financial assets when compared to the non-participating group. An additional limitation is that since the SHARE dataset is not connected directly to an objective patient medical database, it is unknown whether individuals categorized as having depression caseness (according to the EURO-D scale) were diagnosed with clinical depression. However, previous studies demonstrated that the EURO-D scale provides a good assessment of developing clinical depressive disorder when using an optimal cutoff point of 4 depressive symptoms or above [43, 48], as was used in this study. The fact that our analysis is based on self-report data not backed by physical evidence can also be viewed as a limitation. Finally, caution is advised when interpreting the connections established in this study. Being informative in characterizing individuals aged 50 + with depression caseness, they do not imply any cause-and-effect relationships between the variables.

## Conclusions

The results of this study suggest that participation in social activities serves as a mediating factor for overcoming the negative effects of depression. In turn, this effect may increase the financial risk-taking intentions and behavior in favor of investing in risky financial assets that have the potential to improve the financial future of 50 + individuals. Considering higher depression rates in this age group caused by COVID-19, it seems important to engage such individuals in social activities in order to overcome their financial risk aversion associated with lack of social engagement. Thus, engaging 50 + individuals in social events can improve their financial well-being. These findings can be utilized by healthcare authorities as the guidelines for updating their policies and encouraging individuals with depression to regularly participate in various social events.

Applying this recommendation to the Israeli context, the Ministry of Health should encourage the HMOs to expand their spheres of influence by funding and facilitating the participation of older adults in social activities, particularly those of educational or training character. This can potentially bring two positive financial outcomes on the national level. First, it may improve the population's mental health, lower their risk of late-life depression, and thus save HMOs’ costs by reducing hospitalization-related expenses. Second, enjoying better mental health, individuals aged 50 and above may become less risk-averse and more willing to channel the funds available to them into the country’s economy.

### Supplementary Information

Additional file 1: 1. SHARE questions used for the paper: 1.1. EURO-D questions. 1.2. Social activities questions. 1.3. Risky financial assets questions. 1.4. Risk aversion question. 1.5. Marital status question. 1.6. Children. 1.7. Average household monthly income. 2. Dependence and connection between participating in social activities and financial risk-taking intentions and behavior in individuals with ≥ 3, ≥ 4, and ≥ 5 depressive symptoms. 3. Dependence and connection between participating in social activities and financial risk-taking intentions and behavior in individuals participating in at least one / at least two social activities. 4. Dependence and connection between participating in social activities and financial risk-taking behavior for participants of all marital status, and participants reporting no relationship. 5. Dependence and connection between participating in social activities and financial risk-taking intentions and behavior among individuals with and without depression caseness, without depression caseness, and with depression caseness. 6. Stratification by gender within age groups: 6.1. Stratification by gender within age groups: Dependence and connection between participating in social activities and financial risk-taking intentions. 6.2. Stratification by gender within age groups: Dependence and connection between participation in social activities and financial risk-taking behavior. 7. Examination of controls in the connection between participation in social activities and financial risk-taking intentions and behavior: 7.1. Social activities and financial risk-taking intentions (controls: gender, age, marital status, number of children and average household monthly income). 7.2. Social activities and investments in stocks or shares (controls: gender, age, marital status, number of children and average household monthly income). 7.3. Social activities and investments in mutual funds or managed investments accounts (controls: gender, age, marital status, number of children and average household monthly income). 7.4. Social activities and investments in at least one risky financial asset (controls: gender, age, marital status, presence of children and average household monthly income). 8. Financial risk-taking intentions and behavior among individuals not participating in social activities, with or without depression caseness.

## Data Availability

SHARE data is free of charge for scientific use globally (available at www.share-eric.eu/data/data-access).

## Abbreviations

COVID-19: Coronavirus disease 2019
HMO: Health maintenance organizations
SHARE: Survey of Health, Ageing and Retirement in Europe
WHO: World Health Organization
CI: Confidence interval
OR: Odds ratio
EUR: Euro
CLD: Compact letter display
ICT: Information and communication technologies

## References

[1] Hammar Å, Årdal G. Cognitive functioning in major depression – a summary. Front Hum Neurosci. 2009;3(26):1–7. 10.3389/neuro.09.026.2009. 19826496 10.3389/neuro.09.026.2009 PMC2759342

[2] National Institute of Mental Health. Major Depression. 2019. Available from: https://www.nimh.nih.gov/health/statistics/major-depression.shtml. Accessed 18 Apr 2024.

[3] Zuckerman H, Pan Z, Park C, Brietzke E, Musial N, Shariq AS, et al. Recognition and Treatment of Cognitive Dysfunction in Major Depressive Disorder. Front Psychiatry. 2018;9(655):1–11. 10.3389/fpsyt.2018.00655. 30564155 10.3389/fpsyt.2018.00655 PMC6288549

[4] World Health Organization. Global burden of mental disorders and the need for a comprehensive, coordinated response from health and social sectors at the country level: Report by the Secretariat. Executive Board (EB130/9) 130th session, Provisional agenda item 6.2. 2011. Available from: https://apps.who.int/gb/ebwha/pdf_files/EB130/B130_9-en.pdf

[5] Xiong J, Lipsitz O, Nasri F, Lui LMW, Gill H, Phan L, et al. Impact of COVID-19 pandemic on mental health in the general population: A systematic review. J Affect Disord. 2020;277:55–64. 10.1016/j.jad.2020.08.001. 32799105 10.1016/j.jad.2020.08.001 PMC7413844

[6] World Health Organization. WHO Director-General’s statement on IHR Emergency Committee on Novel Coronavirus (2019-nCoV). 2020. Available from: https://www.who.int/director-general/speeches/detail/who-director-general-s-statement-on-ihr-emergency-committee-on-novel-coronavirus-(2019-ncov). Accessed 18 Apr 2024.

[7] Bialek S, Boundy E, Bowen V, Chow N, Cohn A, Dowling N, et al. Severe Outcomes Among Patients with Coronavirus Disease 2019 (COVID-19) — United States, February 12–March 16, 2020. MMWR Morb Mortal Wkly Rep. 2020;69(12):343–6. 10.15585/mmwr.mm6912e2. 32214079 10.15585/mmwr.mm6912e2 PMC7725513

[8] Raina P, Wolfson C, Griffith L, Kirkland S, McMillan J, Basta N, et al. A longitudinal analysis of the impact of the COVID-19 pandemic on the mental health of middle-aged and older adults from the Canadian longitudinal study on aging. Nat Aging. 2021;1(12):1137–47. 10.1038/s43587-021-00128-1. 37117519 10.1038/s43587-021-00128-1

[9] Levasseur M, Richard L, Gauvin L, Raymond É. Inventory and analysis of definitions of social participation found in the aging literature: proposed taxonomy of social activities. Soc Sci Med. 2010;71(12):2141–9. 10.1016/j.socscimed.2010.09.041. 21044812 10.1016/j.socscimed.2010.09.041 PMC3597625

[10] Isaac V, Stewart R, Artero S, Ancelin ML, Ritchie K. Social activity and improvement in depressive symptoms in older people: a prospective community cohort study. Am J Geriatr Psychiatry. 2009;17(8):688–96. 10.1097/JGP.0b013e3181a88441. 19625786 10.1097/JGP.0b013e3181a88441

[11] Bath PA, Deeg D. Social engagement and health outcomes among older people: introduction to a special section. Eur J Ageing. 2005;2(1):24–30. 10.1007/s10433-005-0019-4. 28794713 10.1007/s10433-005-0019-4 PMC5547666

[12] von Bonsdorff MB, Rantanen T. Benefits of formal voluntary work among older people. A review. Aging Clin Exp Res. 2011;23(3):162–9. 10.3275/7200. 20647739 10.3275/7200

[13] Croezen S, Haveman-Nies A, Alvarado VJ, Veer P, Groot CPGM. Characterization of different groups of elderly according to social engagement activity patterns. J Nutr Heal Aging. 2009;13(9):776–81. 10.1007/s12603-009-0213-8.10.1007/s12603-009-0213-819812867

[14] Croezen S, Avendano M, Burdorf A, Van Lenthe FJ. Social Participation and depression in old age: a fixed-effects analysis in 10 European countries. Am J Epidemiol. 2015;182(2):168–76. 10.1093/aje/kwv015. 26025236 10.1093/aje/kwv015 PMC4493978

[15] Glass TA, De Leon CFM, Bassuk SS, Berkman LF. Social engagement and depressive symptoms in late life: longitudinal findings. J Aging Health. 2006;18(4):604–28. 10.1177/0898264306291017. 16835392 10.1177/0898264306291017

[16] Gleibs IH, Haslam C, Jones JM, Alexander Haslam S, McNeill J, Connolly H. No country for old men? The role of a ‘Gentlemen’s Club’ in promoting social engagement and psychological well-being in residential care. Aging Ment Health. 2011;15(4):456–66. 10.1080/13607863.2010.536137. 21500012 10.1080/13607863.2010.536137

[17] Lindström M, Moghaddassi M, Merlo J. Individual self-reported health, social participation and neighbourhood: a multilevel analysis in Malmö. Sweden Prev Med. 2004;39(1):135–41. 10.1016/j.ypmed.2004.01.011. 15207994 10.1016/j.ypmed.2004.01.011

[18] Alloy LB, Ahrens AH. Depression and pessimism for the future: Biased use of statistically relevant information in predictions for self versus others. J Pers Soc Psychol. 1987;52(2):366–78. 10.1037/0022-3514.52.2.366. 3559896 10.1037/0022-3514.52.2.366

[19] Henriques JB, Davidson RJ. Decreased responsiveness to reward in depression. Cogn Emot. 2000;14(5):711–24. 10.1080/02699930050117684. 10.1080/02699930050117684

[20] Pizzagalli DA, Iosifescu D, Hallett LA, Ratner KG, Fava M. Reduced Hedonic Capacity in Major Depressive Disorder: Evidence from a Probabilistic Reward Task. J Psychiatr Res. 2008;43(1):76–87. 10.1016/j.jpsychires.2008.03.001. 18433774 10.1016/j.jpsychires.2008.03.001 PMC2637997

[21] Mata R, Frey R, Richter D, Schupp J, Hertwig R. Risk preference: a view from psychology. J Econ Perspect. 2018;32(2):155–72. 10.1257/jep.32.2.155. 30203934 10.1257/jep.32.2.155

[22] Cobb-Clark DA, Dahmann SC, Kettlewell N. Depression, risk preferences, and risk-taking behavior. J Hum Resour. 2022;57(5):1566–604. 10.3368/jhr.58.1.0419-10183R1. 10.3368/jhr.58.1.0419-10183R1

[23] Dohmen T, Falk A, Golsteyn BHH, Huffman D, Sunde U. Risk attitudes across the life course. Econ J. 2017;127(605):F95-116. 10.1111/ecoj.12322. 10.1111/ecoj.12322

[24] Dohmen T, Falk A, Huffman D, Sunde U, Schupp J, Wagner GG. Individual risk attitudes: measurement, determinants, and behavioral consequences. J Eur Econ Assoc. 2011;9(3):522–50. 10.1111/j.1542-4774.2011.01015.x. 10.1111/j.1542-4774.2011.01015.x

[25] Beauchamp JP, Cesarini D, Johannesson M. The psychometric and empirical properties of measures of risk preferences. J Risk Uncertain. 2017;54(3):203–37. 10.1007/s11166-017-9261-3. 10.1007/s11166-017-9261-3

[26] Kirk L, Haaga DAF, Solomon A, Brody C. Perceptions of depression among never-depressed and recovered-depressed people. Cognit Ther Res. 2000;24(5):585–94. 10.1023/A:1005518229707. 10.1023/A:1005518229707

[27] Smoski MJ, Lynch TR, Rosenthal MZ, Cheavens JS, Chapman AL, Krishnan RR. Decision-making and risk aversion among depressive adults. J Behav Ther Exp Psychiatry. 2008;39(4):567–76. 10.1016/j.jbtep.2008.01.004. 18342834 10.1016/j.jbtep.2008.01.004 PMC2590786

[28] Lindeboom M, Melnychuk M. Mental health and asset choices. Ann Econ Stat. 2015;(119/120):65–94. 10.15609/annaeconstat2009.119-120.65.

[29] Melnychuk M. Depression and willingness to invest in risky financial assets. J Ment Health Policy Econ. 2013;16:S24–S24.

[30] Permanent Mission of Israel to the United Nations and Other International Organizations in Geneva. Mainstreaming Ageing in Israel. 2020. Available from: https://unece.org/sites/default/files/2021-03/Israel_CN_EN.pdf

[31] Health expenditure in Israel - an international comparison of demographic factors and cost structure. BANK OF ISRAEL Office of the Spokesman and Economic Information. 2013;1–4. Available from: https://boi.org.il/en/communication-and-publications/press-releases/excerpt-from-recent-economic-developments-to-be-published-shortly/. Accessed 18 Apr 2024.

[32] Tucker N. Israel is getting older: what do you do in the world of advertising when the audience is over 50? [Hebrew]. TheMarker. 2012. Available from: https://www.themarker.com/advertising/2012-08-26/ty-article/0000017f-ebd9-d639-af7f-ebdf9c070000. Accessed 18 Apr 2024.

[33] Debowy M, Epstein G, Weiss A. Top Decile Wage Earners in Israel. Taub Center for Social Policy Studies in Israel. 2020. Available from: https://www.taubcenter.org.il/wp-content/uploads/2022/05/Top-Decile-Wage-Earners-in-Israel-ENG.pdf

[34] Börsch-Supan A, Brugiavini A, Jürges H, Kapteyn A, Mackenbach J, Siegrist J, et al., editors. First Results from the Survey of Health, Ageing and Retirement in Europe (2004–2007). Starting the Longitudinal Dimension. Mannheim: Mannheim Research Institute for the Economics of Aging (MEA). 2008:364. Available from: https://share-eric.eu/fileadmin/user_upload/First_Results_Books/FRB2_all_chapters.pdf

[35] Börsch-Supan A. Release version: 7.1.0. SHARE-ERIC. Data set. Survey of Health, Ageing and Retirement in Europe (SHARE) Wave 2. 2020. 10.6103/SHARE.w2.710.

[36] Bergmann M, Kneip T, De Luca G, Scherpenzeel A. Survey participation in the Survey of Health, Ageing and Retirement in Europe (SHARE), Wave 1–7. Based on Release 7.0.0. SHARE Working Paper Series 41–2019. Munich: MEA, Max Planck Institute for Social Law and Social Policy. 2019.

[37] SHARE. WAVE 2. Survey of Health, Ageing and Retirement in Europe. 2024. Available from: https://www.share-eric.eu/data/data-documentation/questionnaires/wave-2. Accessed 18 Apr 2024.

[38] SHARE. WAVE 4. Survey of Health, Ageing and Retirement in Europe. 2024. Available from: https://www.share-eric.eu/data/data-documentation/questionnaires/wave-4. Accessed 18 Apr 2024.

[39] Börsch-Supan A, Brandt M, Hunkler C, Kneip T, Korbmacher J, Malter F, et al. Data resource profile: The Survey of Health, Ageing and Retirement in Europe (SHARE). Int J Epidemiol. 2013;42(4):992–1001. 10.1093/ije/dyt088. 23778574 10.1093/ije/dyt088 PMC3780997

[40] Bower JL, Paine LS. The error at the heart of corporate leadership. Harv Bus Rev. 2017;95(3):50–60.

[41] Maloney T, Almeida R. Lengthening the Investment Time Horizon. MFS Investment Management White Paper. 2019; Available from: https://globalfundsearch.com/wp-content/uploads/2019/12/Lengthening-the-Investment-Time-Horizon.pdf

[42] Haldane A. Patience and finance. Bank Int Settlements Rev. 2010;114:1–21. Available from: https://www.bis.org/review/r100909e.pdf

[43] Prince MJ, Reischies F, Beekman ATF, Fuhrer R, Jonker C, Kivela SL, et al. Development of the EURO-D scale - a European Union initiative to compare symptoms of depression in 14 European centres. Br J Psychiatry. 1999;174(4):330–8. 10.1192/bjp.174.4.330. 10533552 10.1192/bjp.174.4.330

[44] Mehrbrodt T, Gruber S, Wagner M. Scales and multi-item indicators. 2021. Available from: https://share-eric.eu/fileadmin/user_upload/Other_Publications/ScalesManual_rel.8-0-0.pdf

[45] World Health Organization. Improving the lives of people with dementia and old-age depression. Regional Office for Europe. 2019. Available from: https://who-sandbox.squiz.cloud/en/countries/netherlands/news2/news/2019/09/improving-the-lives-of-people-with-dementia-and-old-age-depression. Accessed 18 Apr 2024.

[46] Guerra M, Ferri C, Llibre J, Prina AM, Prince M. Psychometric properties of EURO-D, a geriatric depression scale: a cross-cultural validation study. BMC Psychiatry. 2015;15(1):12. 10.1186/s12888-015-0390-4. 25652111 10.1186/s12888-015-0390-4 PMC4332422

[47] Castro-Costa E, Dewey M, Stewart R, Banerjee S, Huppert F, Mendonca-Lima C, et al. Prevalence of depressive symptoms and syndromes in later life in ten European countries: The SHARE study. Br J Psychiatry. 2007;191(5):393–401. 10.1192/bjp.bp.107.036772. 17978318 10.1192/bjp.bp.107.036772

[48] Castro-Costa E, Dewey M, Stewart R, Banerjee S, Huppert F, Mendonca-Lima C, et al. Ascertaining late-life depressive symptoms in Europe: an evaluation of the survey version of the EURO-D scale in 10 nations. The SHARE project. Int J Methods Psychiatr Res. 2008;17(1):12–29. 10.1002/mpr.236. 18286461 10.1002/mpr.236 PMC6878368

[49] Dewey ME, Prince MJ. Mental health. In: Börsch-Supan A, Jürges H, editors. Health, Ageing and Retirement in Europe – First Results from the Survey of Health, Ageing and Retirement in Europe. Mannheim: Mannheim Research Institute for the Economics of Aging (MEA); 2005:108–117.

[50] Arpino B, Bordone V. Regular provision of grandchild care and participation in social activities. Rev Econ Househ. 2017;15(1):135–74. 10.1007/s11150-016-9322-4. 10.1007/s11150-016-9322-4

[51] Christelis D, Jappelli T, Padula M. Cognitive abilities and portfolio choice. Eur Econ Rev. 2010;54(1):18–38. 10.1016/j.euroecorev.2009.04.001. 10.1016/j.euroecorev.2009.04.001

[52] Love DA, Smith PA. Does health affect portfolio choice? Health Econ. 2010;19(12):1441–60. 10.1002/hec.1562. 19937612 10.1002/hec.1562

[53] SHARE. Release Guide 8.0.0. 2022. Available from: https://share-eric.eu/fileadmin/user_upload/Release_Guides/SHARE_release_guide_8-0-0.pdf

[54] Council of Europe. Gender Matters - Glossary. 2024. Available from: https://www.coe.int/en/web/gender-matters/glossary. Accessed 18 Apr 2024.

[55] Curtin R, Presser S, Singer E. The effects of response rate changes on the index of consumer sentiment. Public Opin Q. 2000;64(4):413–28. 10.1086/318638. 11171024 10.1086/318638

[56] Singer E, Van Hoewyk J, Maher MP. Experiments with incentives in telephone surveys. Public Opin Q. 2000;64(2):171–88. 10.1086/317761. 10984332 10.1086/317761

[57] Albert PR. Why is depression more prevalent in women? J Psychiatry Neurosci. 2015;40(4):219–21. 10.1503/jpn.150205. 26107348 10.1503/jpn.150205 PMC4478054

[58] Elliott SA. Postnatal depression. In: Cambridge Handbook of Psychology, Health and Medicine, Second Edition. Cambridge University Press; 2014. p. 820–3. 10.1017/CBO9780511543579.214.

[59] Holkar M. Seeing through the FOG. The Money and Mental Health Policy Institute. 2017. Available from: http://www.moneyandmentalhealth.org/wp-content/uploads/2017/02/Seeing-through-the-fog-Final-report-1.pdf

[60] Smoski MJ, Felder J, Bizzell J, Green SR, Ernst M, Lynch TR, et al. fMRI of alterations in reward selection, anticipation, and feedback in major depressive disorder. J Affect Disord. 2009;118(1–3):69–78. 10.1016/j.jad.2009.01.034. 19261334 10.1016/j.jad.2009.01.034 PMC2745481

[61] National Research Council. Chapter 7: Saving and retirement security & Chapter 8: Capital markets and rates of return. In: Aging and the macroeconomy: long-term implications of an older population. Washington, DC: The National Academies Press;2012. p. 122–73. 10.17226/13465.

[62] Treadway MT, Bossaller N, Shelton RC, Zald DH. Effort-based decision-making in major depressive disorder: a translational model of motivational anhedonia. J Abnorm Psychol. 2012;121(3):553–8. 10.1037/a0028813. 22775583 10.1037/a0028813 PMC3730492

[63] Powell M, Ansic D. Gender differences in risk behaviour in financial decision-making: An experimental analysis. J Econ Psychol. 1997;18(6):605–28. 10.1016/S0167-4870(97)00026-3. 10.1016/S0167-4870(97)00026-3

[64] Nelson JA. Are women really more risk-averse than men? A re-analysis of the literature using expanded methods. J Econ Surv. 2015;29(3):566–85. 10.1111/joes.12069. 10.1111/joes.12069

[65] Jianakoplos NA, Bernasek A. Are women more risk averse? Econ Inq. 1998;36(4):620–30. 10.1111/j.1465-7295.1998.tb01740.x. 10.1111/j.1465-7295.1998.tb01740.x

[66] Bayyurt N, Vildan K, Coşkun A. Gender Differences in Investment Preferences. Eur J Econ Polit Stud Gend. 2013;6(1):71–83. Available from: https://www.researchgate.net/profile/Ali-Coskun-5/publication/277313389_Gender_Differences_in_Investment_Preferences/links/556760ae08aec2268300fc3f/Gender-Differences-in-Investment-Preferences.pdf.

[67] Kliber A, Łęt B, Rutkowska A. Socio-demographic characteristics of investors in the Warsaw Stock Exchange-How they influence the investment decision. Bank i Kredyt. 2016;47(2):91–118. Available from: https://bankikredyt.nbp.pl/content/2016/02/bik_02_2016_01_art.pdf.

[68] Brooks C, Sangiorgi I, Hillenbrand C, Money K. Why are older investors less willing to take financial risks? Int Rev Financ Anal. 2018;56:52–72. 10.1016/j.irfa.2017.12.008. 10.1016/j.irfa.2017.12.008

[69] Vrieze E, Pizzagalli DA, Demyttenaere K, Hompes T, Sienaert P, de Boer P, et al. Reduced Reward Learning Predicts Outcome in Major Depressive Disorder. Biol Psychiatry. 2013;73(7):639–45. 10.1016/j.biopsych.2012.10.014. 23228328 10.1016/j.biopsych.2012.10.014 PMC3602158

[70] Jang Y, Chiriboga DA. Social activity and depressive symptoms in Korean American older adults: the conditioning role of acculturation. J Aging Health. 2011;23(5):767–81. 10.1177/0898264310396214. 21273501 10.1177/0898264310396214 PMC5788311

[71] Saez E, Zucman G. Wealth Inequality in the United States since 1913: evidence from capitalized income tax data. Q J Econ. 2016;131(2):519–78. 10.1093/qje/qjw004. 10.1093/qje/qjw004

[72] Vandenbroucke G, Zhu H. Aging and wealth inequality. Econ Synopses. 2017;(2):1–2. 10.20955/es.2017.2.

[73] Vozikaki M, Linardakis M, Micheli K, Philalithis A. Activity participation and well-being among European adults aged 65 years and older. Soc Indic Res. 2017;131(2):769–95. 10.1007/s11205-016-1256-y. 10.1007/s11205-016-1256-y

[74] Hilary G, Hui KW. Does religion matter in corporate decision making in America? J financ econ. 2009;93(3):455–73. 10.1016/j.jfineco.2008.10.001. 10.1016/j.jfineco.2008.10.001

[75] Jiang F, Jiang Z, Kim KA, Zhang M. Family-firm risk-taking: Does religion matter? J Corp Financ. 2015;33:260–78. 10.1016/j.jcorpfin.2015.01.007. 10.1016/j.jcorpfin.2015.01.007

[76] Kanagaretnam K, Lobo GJ, Wang C, Whalen DJ. Religiosity and risk-taking in international banking. J Behav Exp Financ. 2015;7:42–59. 10.1016/j.jbef.2015.07.004. 10.1016/j.jbef.2015.07.004

[77] Malinowski B. Magic, science and religion. In: Needham J, editor. Science, Religion and Reality. New York: The Macmillan company; 1925. p. 19–94. 10.5962/bhl.title.47892.

[78] Homans GC. Anxiety and Ritual: The theories of Malinowski and Radcliffe-Brown. Am Anthropol. 1941;43(2):164–72. Available from: https://www.jstor.org/stable/662949. 10.1525/aa.1941.43.2.02a00020

[79] Engstrom HR, Laurin K. Chapter 17 - Existential uncertainty and religion. In: Vail KE, Routledge C, editors. The Science of Religion, Spirituality, and Existentialism. Elsevier; 2020. p. 243–59. 10.1016/B978-0-12-817204-9.00018-4.

[80] Jin B. The impact of participation in educational programs on elderly’s life satisfaction. In: Adult Education Research Conference. 2017. p. 1–10. Available from: https://newprairiepress.org/aerc/2017/papers/18.

[81] Hebestreit L. The role of the university of the third age in meeting needs of adult learners in Victoria, Australia. Aust J Adult Learn. 2008;48(3):547–65. Available from: https://files.eric.ed.gov/fulltext/EJ828997.pdf.

[82] Shapira N, Barak A, Gal I. Promoting older adults’ well-being through Internet training and use. Aging Ment Health. 2007;11(5):477–84. 10.1080/13607860601086546. 17882585 10.1080/13607860601086546

[83] Guido G, Amatulli C, Sestino A. Elderly consumers and financial choices: A systematic review. J Financ Serv Mark. 2020;25(3–4):76–85. 10.1057/s41264-020-00077-7. 10.1057/s41264-020-00077-7

[84] Masud J, Haron SA, Gikonyo LW. Gender differences in income sources of the elderly in Peninsular Malaysia. J Fam Econ Issues. 2008;29(4):623–33. 10.1007/s10834-008-9125-8. 10.1007/s10834-008-9125-8

