# Supplementary material for: Exploring the relationship between social activities and financial risk aversion in adults aged 50 + with depression caseness
Source: Isr J Health Policy Res. 2024 Jul 30;13:34. doi: 10.1186/s13584-024-00621-z (PMC11290178; doi:10.1186/s13584-024-00621-z)
Supplement: Supplementary file 1 — Additional file 1: 1. SHARE questions used for the paper: 1.1. EURO-D questions. 1.2. Social activities questions. 1.3. Risky financial assets questions. 1.4. Risk aversion question. 1.5. Marital status question. 1.6. Children. 1.7. Average household monthly income. 2. Dependence and connection between participating in social activities and financial risk-taking intentions and behavior in individuals with ≥ 3, ≥ 4, and ≥ 5 depressive symptoms. 3. Dependence and connection between participating in social activities and financial risk-taking intentions and behavior in individuals participating in at least one / at least two social activities. 4. Dependence and connection between participating in social activities and financial risk-taking behavior for participants of all marital status, and participants reporting no relationship. 5. Dependence and connection between participating in social activities and financial risk-taking intentions and behavior among individuals with and without depression caseness, without depression caseness, and with depression caseness. 6. Stratification by gender within age groups: 6.1. Stratification by gender within age groups: Dependence and connection between participating in social activities and financial risk-taking intentions. 6.2. Stratification by gender within age groups: Dependence and connection between participation in social activities and financial risk-taking behavior. 7. Examination of controls in the connection between participation in social activities and financial risk-taking intentions and behavior: 7.1. Social activities and financial risk-taking intentions (controls: gender, age, marital status, number of children and average household monthly income). 7.2. Social activities and investments in stocks or shares (controls: gender, age, marital status, number of children and average household monthly income). 7.3. Social activities and investments in mutual funds or managed investments accounts (controls: gender, age, m [file 13584_2024_621_MOESM1_ESM.docx]

**Supplementary Information**

**Exploring the relationship between social activities and financial risk aversion in adults aged 50+ with depression caseness**

**Shay Musbat^1^, Inbal Reuveni^2^ and Racheli Magnezi^1*^**

^1^Department of Management, Health Systems Management Program, Bar-Ilan University, Ramat Gan 5290002, Israel. ^2^Department of Psychiatry, Hadassah Hebrew University Medical Center, Ein Kerem, Jerusalem 9112001, Israel.

*Correspondence: Email: [Racheli.Magnezi@biu.ac.il](mailto:Racheli.Magnezi@biu.ac.il).

**1. SHARE questions used for the paper:**

The questions used for the paper were taken from the SHARE (Survey of Health, Ageing and Retirement in Europe) Wave 2. SHARE Wave 2 questionnaire is available online: <https://share-eric.eu/fileadmin/user_upload/Questionnaires/Q-Wave_2/w2_en_capi_main-Generic.pdf>. The letters and numbers combination in the parentheses is the question code used by the SHARE questionnaire.

**1.1.** **EURO-D questions:**

EURO depression scale

“*SHARE provides the EURO-D variable (eurod) and the EURO-D caseness variable (eurodcat) as generated variables in the gv_health module. eurod is generated from questions in the mental health module (mh002_ – mh017_) as a composite index of the sixteen items. Please note that information in mh005_/mh006_, mh008_/mh009_, mh011_/mh012_ and mh014_/mh015_ is combined when generating eurod so that the list of 16 items is reduced to 12 final items. The maximum score a respondent can get is 12 ‘very depressed’ and the minimum score is 0 ‘not depressed’. The attainment of a scale score of 4 or higher is categorized as ‘case of depression’ and a scale score below 4 as ‘not depressed’. The generated variable eurodcat equals 1 if the scale score is 4 or higher*.” (1 p3).

In each EURO-D question, the categories -2. Refusal, -1. Don’t know, 0. Not selected, 1. Selected, were applied according to participant answer.

1) DEPRESSION (MH002_) - Euro1 - Depression

Question

In the last month, have you been sad or depressed?

*IWER: IF PARTICIPANT ASKS FOR CLARIFICATION, SAY 'BY SAD OR DEPRESSED, WE MEAN MISERABLE, IN LOW SPIRITS, OR BLUE'*

Response options:

1. Yes

5. No

2) HOPES FOR THE FUTURE (MH003_) - Euro2 - Pessimism

Question

What are your hopes for the future?

*IWER: NOTE ONLY WHETHER HOPES ARE MENTIONED OR NOT*

Response options:

1. Any hopes mentioned

2. No hopes mentioned

3) FELT WOULD RATHER BE DEAD (MH004_) - Euro3 - Suicidality

Question

In the last month, have you felt that you would rather be dead?

Response options:

1. Any mention of suicidal feelings or wishing to be dead

2. No such feelings

4) FEELS GUILTY (MH005_) - Euro4 1/2 - Guilt

Question

Do you tend to blame yourself or feel guilty about anything?

Response options:

1. Obvious excessive guilt or self-blame

2. No such feelings

3. Mentions guilt or self-blame, but it is unclear if these constitute obvious or excessive guilt or self-blame

*IF MH005_ (FEELS GUILTY) = 3. Mentions guilt or self-blame, but it is unclear if these constitute obvious or excessive guilt or self-blame*

5) BLAME FOR WHAT (MH006_) - Euro4 2/2 – Guilt

Question

So, for what do you blame yourself?

*IWER: NOTE - ONLY CODE 1 FOR AN EXAGGERATED FEELING OF GUILT, WHICH IS CLEARLY OUT OF PROPORTION TO THE CIRCUMSTANCES. THE FAULT WILL OFTEN HAVE BEEN VERY MINOR, IF THERE WAS ONE AT ALL. JUSTIFIABLE OR APPROPRIATE GUILT SHOULD BE CODED 2.*

Response options:

1. Example(s) given constitute obvious excessive guilt or self-blame

2. Example(s) do not constitute obvious excessive guilt or self-blame, or it remains unclear if these constitute obvious or excessive guilt or self-blame

6) TROUBLE SLEEPING (MH007_) - Euro5 - Sleep

Question

Have you had trouble sleeping recently?

Response options:

1. Trouble with sleep or recent change in pattern

2. No trouble sleeping

7) LESS OR SAME INTEREST IN THINGS (MH008_) - Euro6 1/2 – Interest

Question

In the last month, what is your interest in things?

Response options:

1. Less interest than usual mentioned

2. No mention of loss of interest

3. Non-specific or uncodeable response

*IF MH008_ (LESS OR SAME INTEREST IN THINGS) = 3. Non-specific or uncodeable response*

8) KEEPS UP INTEREST (MH009_) - Euro6 2/2 - Interest

Question

So, do you keep up your interests?

Response options:

1. Yes

5. No

9) IRRITABILITY (MH010_) - Euro7 - Irritability

Question

Have you been irritable recently?

Response options:

1. Yes

5. No

10) APPETITE (MH011_) - Euro8 1/2 - Appetite

Question

What has your appetite been like?

Response options:

1. Diminution in desire for food

2. No diminution in desire for food

3. Non-specific or uncodeable response

*IF MH011_ (APPETITE) = 3. Non-specific or uncodeable response*

11) EATING MORE OR LESS (MH012_) - Euro8 2/2 - Appetite

Question

So, have you been eating more or less than usual?

Response options:

1. Less

2. More

3. Neither more nor less

12) FATIGUE (MH013_) - Euro9 – Fatigue

Question

In the last month, have you had too little energy to do the things you wanted to do?

Response options:

1. Yes

5. No

13) CONCENTRATION ON ENTERTAINMENT (MH014_) - Euro10 1/2 - Concentration

Question

How is your concentration? For example, can you concentrate on a television programme, film or radio programme?

Response options:

1. Difficulty in concentrating on entertainment

2. No such difficulty mentioned

14) CONCENTRATION ON READING (MH015_) - Euro10 2/2 - Concentration

Question

Can you concentrate on something you read?

Response options:

1. Difficulty in concentrating on reading

2. No such difficulty mentioned

15) ENJOYMENT (MH016_) - Euro11 - Enjoyment

Question
What have you enjoyed doing recently?

Response options:

1. Fails to mention any enjoyable activity

2. Mentions ANY enjoyment from activity

16) TEARFULNESS (MH017_) - Euro12 - Tearfulness

Question

In the last month, have you cried at all?

Response options:

1. Yes

5. No

**1.2.** **Social activities questions:**

ACTIVITIES IN LAST MONTH (AC002_)

Question

Please look at card 48. Have you done any of these activities in the last month?

*IWER: CODE ALL THAT APPLY. TAKING PART IN ACTIVITIES OF A RELIGIOUS ORGANIZATION INCLUDES CHURCH, SYNAGOGUE, MOSQUE ATTENDANCE.*

1. Done voluntary or charity work (ac002d1)

2. Cared for a sick or disabled adult (ac002d2)

3. Provided help to family, friends or neighbors (ac002d3)

4. Attended an educational or training course (ac002d4)

5. Gone to a sport, social or other kind of club (ac002d5)

6. Taken part in activities of a religious organization (church, synagogue, mosque etc.) (ac002d6)

7. Taken part in a political or community-related organization (ac002d7)

96. None of these (ac002dno)

Response options:

-2. Refusal

-1. Don’t know

0. Not selected

1. Selected

*LOOP cnt1:= 1 TO 7*

*IF cnt1 IN AC002_(ACTIVITIES IN LAST MONTH)*

HOW OFTEN ACTIVITY IN THE LAST FOUR WEEKS (AC003_)

Question

How often in the last four weeks [did/have you]

1. Do voluntary or charity work (ac003_1)

2. Cared for a sick or disabled adult (ac003_2)

3. Provided help to friends or neighbors (ac003_3)

4. Attended an educational or training course (ac003_4)

5. Gone to a sport, social or other kind of club (ac003_5)

6. Taken part in activities of a religious organization (church, synagogue, mosque etc.) (ac003_6)

7. Taken part in a political or community-related organization (ac003_7)

Response options:

-2. Refusal

-1. Don’t know

1. Almost daily

2. Almost every week

3. Less often

MOTIVATIONS (AC004_)

Question

Please look at card 49. For which on the reasons given on this card, if any, [did/have you]

1. Do voluntary or charity work

2. Cared for a sick or disabled adult

3. Provided help to friends or neighbors

4. Attended an educational or training course

5. Gone to a sport, social or other kind of club

6. Taken part in activities of a religious organization (church, synagogue, mosque etc.)

7. Taken part in a political or community-related organization

*IWER: CODE ALL THAT APPLY*

1. To meet other people (ac004d1_1-7)

2. To contribute something useful (ac004d2_1-7)

3. Because I am needed (ac004d4_1-7)

4. To earn money (ac004d5_1-7)

5. To use my skills or to keep fit (ac004d7_1-7)

96. None of these (ac004dno_1-7)

Response options:

-2. Refusal

-1. Don’t know

0. Not selected

1. Selected

**1.3. Risky financial assets questions:**

HAS STOCKS (AS063_)

Question

Do you [or your/husband/wife/partner] currently have any money in stocks or shares (listed or unlisted on stockmarket)?

*IWER: STOCKS ARE PIECES OF PAPER THAT SHOW THAT THE PERSON OWNS PART OF A CORPORATION AND HAS THE RIGHT TO RECEIVE DIVIDENDS FROM IT.*

Response options:

-2. Refusal

-1. Don’t know

1. Yes

5. No

HAS MUTUAL FUNDS (AS064_)

Question

Do you [or your husband/wife/partner] currently have any money in mutual funds or managed investment accounts?

*IWER: MUTUAL FUNDS ARE A POOL OF MONEY BELONGING TO MANY INVESTORS WHO TRUST A MANAGER TO INVEST IT IN STOCKS AND/OR BONDS.*

Response options:

-2. Refusal

-1. Don’t know

1. Yes

5. No

**1.4. Risk aversion question:**

RISK AVERSION (AS068_)

Question

Please look at card 46. When people invest their savings they can choose between assets that give low return with little risk to lose money, for instance a bank account or a safe bond, or assets with a high return but also a higher risk of losing, for instance stocks and shares. Which of the statements on the card comes closest to the amount of financial risk that you are willing to take when you save or make investments?

*IWER: READ ANSWERS ONLY IF NECESSARY. IF MORE THAN ONE RESPONSE IS GIVEN USE THE FIRST CATEGORYTHAT APPLIES*

Response options:

-2. Refusal

-1. Don't know

1. Take substantial financial risks expecting to earn substantial returns

2. Take above average financial risks expecting to earn above average returns

3. Take average financial risks expecting to earn average returns

4. Not willing to take any financial risks

**1.5.** **Marital status question:**

MARITAL STATUS (DN014_)

Question

Please look at card 4. What is your marital status?

Response options*:

1. Married and living together with spouse

2. Registered partnership

3. Married, living separated from spouse

4. Never married

5. Divorced

6. Widowed

*The responses were analyzed dichotomously, with options 1 and 2 corresponding to “With relationship”, and options 3-6 corresponding to “Without relationship”.

**1.6. Children**

NUMBER OF CHILDREN (CH001_)

Question

Now I will ask some questions about your children. How many children do you have that are still alive? Please count all natural children, fostered, adopted and stepchildren [including those of your husband/your wife/your partner/your partner].

Response option*:

Number

* To analyze the presence of children, “0” corresponds to “Without children” while any other number corresponds to “With children”.

**1.7. Average household monthly income**

TOTAL INCOME RECEIVED BY ALL HOUSEHOLD MEMBERS IN LAST MONTH (HH017_)

Question

To summarize, how much was the overall income, after tax, that your entire household had in an average month in [previous year]?

Response option:

Amount*

* “*All answers about an amount of money are converted into Euro values.*” (2 p17). Values were divided by 1000.

**2. Dependence and connection between participating in social activities and financial risk-taking intentions and behavior in individuals with** ≥ **3,** ≥ **4, and** ≥ **5 depressive symptoms:**

| **Parameter** | **Risky financial assets** | **Participants** | **Odds ratio and Pearson chi-square** |
| --- | --- | --- | --- |
| Financial risk-taking intentions | - | ≥ 3 depressive symptoms | OR = 2.70, 95% CI: 2.41-3.01, *p* < 0.001*  *χ*^2^(1) = 315.04, *p* < 0.001* |
|  |  | ≥ 4 depressive symptoms | OR = 2.73, 95% CI: 2.37-3.15, *p* < 0.001*  *χ*2(1) = 200.13, p < 0.001* |
|  |  | ≥ 5 depressive symptoms | OR = 2.51, 95% CI: 2.09-3.02, *p* < 0.001*  *χ*^2^(1) = 100.76, *p* < 0.001* |
| Financial risk-taking behavior | Stocks or shares | ≥ 3 depressive symptoms | OR = 3.41, 95% CI: 2.96-3.93, *p* < 0.001*  *χ*^2^(1) = 318.96, *p* < 0.001* |
|  |  | ≥ 4 depressive symptoms | OR = 3.40, 95% CI: 2.82-4.10, *p* < 0.001*  *χ*^2^(1) = 180.32, *p* < 0.001* |
|  |  | ≥ 5 depressive symptoms | OR = 3.03, 95% CI: 2.36-3.88, *p* < 0.001*  *χ*^2^(1) = 82.56, *p* < 0.001* |
|  | Mutual funds or managed investments accounts | ≥ 3 depressive symptoms | OR = 3.53, 95% CI: 3.00-4.15, *p* < 0.001*  *χ*^2^(1) = 258.87, *p* < 0.001* |
|  |  | ≥ 4 depressive symptoms | OR = 3.60, 95% CI: 2.90-4.47, *p* < 0.001*  *χ*^2^(1) = 151.12, *p* < 0.001* |
|  |  | ≥ 5 depressive symptoms | OR = 4.30, 95% CI: 3.23-5.72, *p* < 0.001*  *χ*^2^(1) = 115.21, *p* < 0.001* |
|  | Both types of risky assets | ≥ 3 depressive symptoms | OR = 4.82, 95% CI: 3.75-6.19, *p* < 0.001*  *χ*^2^(1) = 181.34, *p* < 0.001* |
|  |  | ≥ 4 depressive symptoms | OR = 4.97, 95% CI: 3.50-7.05, *p* < 0.001*  *χ*^2^(1) = 97.97, *p* < 0.001* |
|  |  | ≥ 5 depressive symptoms | OR = 4.38, 95% CI: 2.76-6.94, *p* < 0.001*  *χ*^2^(1) = 46.60, *p* < 0.001* |

* Significant.

**3.** **Dependence and connection between participating in social activities and financial risk-taking intentions and behavior in individuals participating in at least one / at least two social activities:**

| **Parameter** | **Risky financial assets** | **Participating in** | **Odds ratio and Pearson chi-square** |
| --- | --- | --- | --- |
| Financial risk-taking intentions | - | At least one social activity | OR = 2.73, 95% CI: 2.37-3.15, *p* < 0.001*  *χ*^2^(1) = 200.13, *p* < 0.001* |
|  |  | At least two social activities | OR = 3.87, 95% CI: 3.24-4.63, *p* < 0.001*  *χ*^2^(1) = 240.79, *p* < 0.001* |
| Financial risk-taking behavior | Stocks or shares | At least one social activity | OR = 3.40, 95% CI: 2.82-4.10, *p* < 0.001*  *χ*^2^(1) = 180.32, *p* < 0.001* |
|  |  | At least two social activities | OR = 5.54, 95% CI: 4.45-6.90, *p* < 0.001*  *χ*^2^(1) = 280.08, *p* < 0.001* |
|  | Mutual funds or managed investments accounts | At least one social activity | OR = 3.60, 95% CI: 2.90-4.47, *p* < 0.001*  *χ*^2^(1) = 151.12, *p* < 0.001* |
|  |  | At least two social activities | OR = 5.64, 95% CI: 4.39-7.24, *p* < 0.001*  *χ*^2^(1) = 223.45, *p* < 0.001* |
|  | Both types of risky assets | At least one social activity | OR = 4.97, 95% CI: 3.50-7.05, *p* < 0.001*  *χ*^2^(1) = 97.97, *p* < 0.001* |
|  |  | At least two social activities | OR = 9.31, 95% CI: 6.34-13.69, *p* < 0.001*  *χ*^2^(1) = 184.07, *p* < 0.001* |

* Significant.

**4. Dependence and connection between participating in social activities and financial risk-taking behavior for participants of all marital status, and participants reporting no relationship:**

| **Risky financial assets** | **Participants** | **Odds ratio and Pearson chi-square** |
| --- | --- | --- |
| Stocks or shares | All marital status | OR = 3.40, 95% CI: 2.82-4.10, *p* < 0.001*  *χ*^2^(1) = 180.32, *p* < 0.001* |
|  | Separated from spouse / never married / divorced / widowed | OR = 3.73, 95% CI: 2.37-5.85, *p* < 0.001*  *χ*^2^(1) = 36.75, *p* < 0.001* |
| Mutual funds or managed investments accounts | All marital status | OR = 3.60, 95% CI: 2.90-4.47, *p* < 0.001*  *χ*^2^(1) = 151.12, *p* < 0.001* |
|  | Separated from spouse / never married / divorced / widowed | OR = 3.64, 95% CI: 2.11-6.27, *p* < 0.001*  *χ*^2^(1) = 24.32, *p* < 0.001* |
| Both types of risky assets | All marital status | OR = 4.97, 95% CI: 3.50-7.05, *p* < 0.001*  *χ*^2^(1) = 97.97, *p* < 0.001* |
|  | Separated from spouse / never married / divorced / widowed divorced / widowed | OR = 11.06, 95% CI: 2.38-51.43, *p* = 0.002*  Fisher's exact test *p <* 0.001* |

* Significant.

**5. Dependence and connection between participating in social activities and financial risk-taking intentions and behavior among individuals with and without depression caseness, without depression caseness, and with depression caseness:**

| **Parameter** | **Risky financial assets** | **Participants** | **Odds ratio and Pearson chi-square** |
| --- | --- | --- | --- |
| Financial risk-taking intentions | - | With and without depression caseness | OR = 2.49, 95% CI: 2.34-2.66, *p* < 0.001*  *χ*^2^(1) = 851.69, *p* < 0.001* |
|  |  | Without depression caseness | OR = 2.29, 95% CI: 2.13-2.45, *p* < 0.001*  *χ*^2^(1) = 549.01, *p* < 0.001* |
|  |  | With depression caseness | OR = 2.73, 95% CI: 2.37-3.15, *p* < 0.001*  *χ*^2^(1) = 200.13, *p* < 0.001* |
| Financial risk-taking behavior | Stocks or shares | With and without depression caseness | OR = 3.20, 95% CI: 2.96-3.46, *p* < 0.001*  *χ*^2^(1) = 917.24, *p* < 0.001* |
|  |  | Without depression caseness | OR = 2.94, 95% CI: 2.69-3.20, *p* < 0.001*  *χ*^2^(1) = 632.58, *p* < 0.001* |
|  |  | With depression caseness | OR = 3.40, 95% CI: 2.82-4.10, *p* < 0.001*  *χ*^2^(1) = 180.32, *p* < 0.001* |
|  | Mutual funds or managed investments accounts | With and without depression caseness | OR = 3.12, 95% CI: 2.86-3.41, *p* < 0.001*  *χ*^2^(1) = 691.31, *p* < 0.001* |
|  |  | Without depression caseness | OR = 2.82, 95% CI: 2.56-3.11, *p* < 0.001*  *χ*^2^(1) = 464.63, *p* < 0.001* |
|  |  | With depression caseness | OR = 3.60, 95% CI: 2.90-4.47, *p* < 0.001*  *χ*^2^(1) = 151.12, *p* < 0.001* |
|  | Both types of risky assets | With and without depression caseness | OR = 4.27, 95% CI: 3.74-4.86, *p* < 0.001*  *χ*^2^(1) = 542.82, *p* < 0.001* |
|  |  | Without depression caseness | OR = 3.81, 95% CI: 3.31-4.39, *p* < 0.001*  *χ*^2^(1) = 384.41, *p* < 0.001* |
|  |  | With depression caseness | OR = 4.97, 95% CI: 3.50-7.05, *p* < 0.001*  *χ*^2^(1) = 97.97, *p* < 0.001* |

* Significant.

**6. Stratification by gender within age groups:**

**6.1.** Stratification by gender within age groups: Dependence and connection between participating in social activities and financial risk-taking intentions.

| **Age group** | **Gender** | **Odds ratio and Pearson chi-square** |
| --- | --- | --- |
| 50-59 | Males and females | OR = 2.60, 95% CI: 2.09-3.24, *p* < 0.001*  *χ*^2^(1) = 74.82, *p* < 0.001* |
|  | Males | OR = 3.47, 95% CI: 2.39-5.03, *p* < 0.001*  *χ*^2^(1) = 44.87, *p* < 0.001* |
|  | Females | OR = 2.27, 95% CI: 1.72-3.00, *p* < 0.001*  *χ*^2^(1) = 34.76, *p* < 0.001* |
| 60-69 | Males and females | OR = 2.19, 95% CI: 1.67-2.86, *p* < 0.001*  *χ*^2^(1) = 33.19, *p* < 0.001* |
|  | Males | OR = 2.20, 95% CI: 1.42-3.43, *p* < 0.001*  *χ*^2^(1) = 12.65, *p* < 0.001* |
|  | Females | OR = 2.32, 95% CI: 1.64-3.29, *p* < 0.001*  *χ*^2^(1) = 23.51, *p* < 0.001* |
| 70-79 | Males and females | OR = 2.25, 95% CI: 1.61-3.15, *p* < 0.001*  *χ*^2^(1) = 23.19, *p* < 0.001* |
|  | Males | OR = 1.77, 95% CI: 1.06-2.96, *p* = 0.030*  *χ*^2^(1) = 4.81, *p* = 0.028* |
|  | Females | OR = 2.74, 95% CI: 1.74-4.31, *p* < 0.001*  *χ*^2^(1) = 20.18, *p* < 0.001* |
| 80+ | Males and females | OR = 2.54, 95% CI: 1.54-4.19, *p* > 0.001*  *χ*^2^(1) = 14.24, *p* < 0.001* |
|  | Males | OR = 2.23, 95% CI: 1.03-4.84, *p* = 0.041*  *χ*^2^(1) = 4.33, *p* = 0.037* |
|  | Females | OR = 2.80, 95% CI: 1.45-5.41, *p* = 0.002*  *χ*^2^(1) = 10.13, *p* = 0.001* |

* Significant

**6.2.** Stratification by gender within age groups: Dependence and connection between participation in social activities and financial risk-taking behavior.

| **Age group** | **Gender** | **Risky financial assets** | **Odds ratio and Pearson chi-square** |
| --- | --- | --- | --- |
| 50-59 | Males and females | Stocks or shares | OR = 3.23, 95% CI: 2.40-4.34, *p* < 0.001*  *χ*^2^(1) = 65.10, *p* < 0.001* |
|  |  | Mutual funds or managed investment accounts | OR = 3.45, 95% CI: 2.41-4.92, *p* < 0.001*  *χ*^2^(1) = 51.29, *p* < 0.001* |
|  |  | Both types of risky assets | OR = 4.01, 95% CI: 2.36-6.80, *p* < 0.001*  *χ*^2^(1) = 30.47, *p* <0.001* |
|  | Males | Stocks or shares | OR = 4.65, 95% CI: 2.79-7.75, *p* < 0.001*  *χ*^2^(1) = 39.15, *p* < 0.001* |
|  |  | Mutual funds or managed investment accounts | OR = 4.20, 95% CI: 2.25-7.85, *p* < 0.001*  *χ*^2^(1) = 23.09, *p* <0.001* |
|  |  | Both types of risky assets | OR = 5.54, 95% CI: 2.48-12.38, *p* < 0.001*  *χ*^2^(1) = 21.12, *p* < 0.001* |
|  | Females | Stocks or shares | OR = 2.69, 95% CI: 1.86-3.87, *p* < 0.001*  *χ*^2^(1) = 29.82, *p* < 0.001* |
|  |  | Mutual funds or managed investment accounts | OR = 3.14, 95% CI: 2.04-4.84, *p* < 0.001*  *χ*^2^(1) = 29.17, *p* < 0.001* |
|  |  | Both types of risky assets | OR = 3.22, 95% CI: 1.59-6.54, *p* = 0.001*  *χ*^2^(1) = 11.67, *p* = 0.001* |
| 60-69 | Males and females | Stocks or shares | OR = 3.38, 95% CI: 2.34-4.88, *p* < 0.001*  *χ*^2^(1) = 46.04, *p* <0.001* |
|  |  | Mutual funds or managed investment accounts | OR = 2.57, 95% CI: 1.74-3.78, *p* < 0.001*  *χ*^2^(1) = 24.23, *p* < 0.001* |
|  |  | Both types of risky assets | OR = 4.35, 95% CI: 2.28-8.30, *p* < 0.001*  *χ*^2^(1) = 23.39, *p* < 0.001* |
|  | Males | Stocks or shares | OR = 2.29, 95% CI: 1.30-4.01, *p* = 0.004*  *χ*^2^(1) = 8.65, *p* = 0.003* |
|  |  | Mutual funds or managed investment accounts | OR = 1.89, 95% CI: 1.01-3.54, *p* = 0.048*  *χ*^2^(1) = 4.01, *p* = 0.045* |
|  |  | Both types of risky assets | OR = 4.86, 95% CI: 1.70-13.89, *p* = 0.003*  *χ*^2^(1) = 10.38, *p* = 0.001* |
|  | Females | Stocks or shares | OR = 4.83, 95% CI: 2.89-8.09, *p* < 0.001*  *χ*^2^(1) = 42.38, *p* < 0.001* |
|  |  | Mutual funds or managed investment accounts | OR = 3.22, 95% CI: 1.94-5.33, *p* < 0.001*  *χ*^2^(1) = 22.59, *p* < 0.001* |
|  |  | Both types of risky assets | OR = 4.19, 95% CI: 1.85-9.51, *p* = 0.001*  *χ*^2^(1) = 13.70, *p* < 0.001* |
| 70-79 | Males and females | Stocks or shares | OR = 2.97, 95% CI: 1.96-4.49, *p* < 0.001*  *χ*^2^(1) = 28.67, *p* < 0.001* |
|  |  | Mutual funds or managed investment accounts | OR = 5.14, 95% CI: 2.95-8.95, *p* < 0.001*  *χ*^2^(1) = 40.34, *p* < 0.001* |
|  |  | Both types of risky assets | OR = 9.56, 95% CI: 3.54-25.78, *p* < 0.001*  *χ*^2^(1) = 29.28, *p* < 0.001* |
|  | Males | Stocks or shares | OR = 2.51, 95% CI: 1.38-4.58, *p* = 0.003*  *χ*^2^(1) = 9.56, *p* = 0.002* |
|  |  | Mutual funds or managed investment accounts | OR = 8.08, 95% CI: 3.17-20.60, *p* < 0.001*  *χ*^2^(1) = 25.73, *p* < 0.001* |
|  |  | Both types of risky assets | OR = 11.68, 95% CI: 3.30-41.43, *p* < 0.001*  *χ*^2^(1) = 22.20, *p* < 0.001* |
|  | Females | Stocks or shares | OR = 3.51, 95% CI: 1.96-6.31, *p* < 0.001*  *χ*^2^(1) = 19.72, *p* < 0.001* |
|  |  | Mutual funds or managed investment accounts | OR = 3.81, 95% CI: 1.89-7.72, *p* < 0.001*  *χ*^2^(1) = 15.81, *p* < 0.001* |
|  |  | Both types of risky assets | OR = 6.30, 95% CI: 1.22-32.67, *p* = 0.028*  Fisher's exact test *p* = 0.023* |
| 80+ | Males and females | Stocks or shares | OR = 2.07, 95% CI: 1.10-3.90, *p* = 0.023*  *χ*^2^(1) = 5.34, *p* = 0.021* |
|  |  | Mutual funds or managed investment accounts | OR = 3.58, 95% CI: 1.99-6.45, *p* < 0.001*  *χ*^2^(1) = 20.21, *p* < 0.001* |
|  |  | Both types of risky assets | OR = 2.03, 95% CI: 0.60-6.80, *p* = 0.253  Fisher's exact test *p* = 0.271 |
|  | Males | Stocks or shares | OR = 2.82, 95% CI: 1.09-7.33, *p* = 0.033*  Fisher's exact test *p* = 0.041* |
|  |  | Mutual funds or managed investment accounts | OR = 2.01, 95% CI: 0.81-4.96, *p* = 0.130  *χ*^2^(1) = 2.36, *p* = 0.124 |
|  |  | Both types of risky assets | OR = 1.65, 95% CI: 0.31-8.75, *p* = 0.555  Fisher's exact test *p* = 0.627 |
|  | Females | Stocks or shares | OR = 1.65, 95% CI: 0.70-3.87, *p* = 0.253  *χ*^2^(1) = 1.33, *p* = 0.248 |
|  |  | Mutual funds or managed investment accounts | OR = 5.93, 95% CI: 2.60-13.51, *p* <0.001*  *χ*^2^(1) = 22.44, *p* < 0.001* |
|  |  | Both types of risky assets | OR = 2.68, 95% CI: 0.44-16.20, *p* = 0.284  Fisher's exact test *p* = 0.264 |

* Significant

**7. Examination of controls in the connection between participation in social activities and financial risk-taking intentions and behavior:**

**7.1.** Social activities and financial risk-taking intentions (controls: gender, age, marital status, number of children and average household monthly income)

| **Variables (Step 1^a^)** | **B** | **S.E.** | **EXP(B) (Odds ratio)** | **95% C.I. for EXP(B)** | **Sig.** |
| --- | --- | --- | --- | --- | --- |
| **Constant** | 0.227 | 0.381 | 1.26 |  | |
| **Gender (male)** | 0.458 | 0.115 | 1.58 | 1.26-1.98 | *p* < 0.001 |
| **Age (Continuous)** | -0.037 | 0.006 | 0.96 | 0.95-0.97 | *p* < 0.001 |
| **Marital status (with relationship)** | 0.128 | 0.115 | 1.14 | 0.91-1.42 | *p* = 0.263 |
| **Number of children (Continuous)** | -0.148 | 0.041 | 0.86 | 0.80-0.93 | *p* < 0.001 |
| **Average household monthly income EUR/1000 (Continuous)** | 0.003 | 0.001 | 1.003 | 1.001-1.004 | *p* < 0.001 |
| **Social activities (with)** | 0.876 | 0.111 | 2.40 | 1.93-2.98 | *p* < 0.001 |

Step 1: Financial risk-taking intentions. a: Variable(s) entered on step 1: Social activities (based on 2,872 individuals who answered all the questions).

**7.2.** Social activities and investments in stocks or shares (controls: gender, age, marital status, number of children and average household monthly income)

| **Variables (Step 1^a^)** | **B** | **S.E.** | **EXP(B) (Odds ratio)** | **95% C.I. for EXP(B)** | **Sig.** |
| --- | --- | --- | --- | --- | --- |
| **Constant** | -2.022 | 0.544 | 0.13 |  | |
| **Gender (male)** | 0.347 | 0.159 | 1.42 | 1.04-1.93 | *p* = 0.029 |
| **Age (Continuous)** | -0.023 | 0.008 | 0.98 | 0.96-0.99 | *p =* 0.003 |
| **Marital status (with relationship)** | 0.226 | 0.158 | 1.25 | 0.92-1.71 | *p =* 0.154 |
| **Number of children (Continuous)** | -0.063 | 0.054 | 0.94 | 0.85-1.04 | *p =* 0.241 |
| **Average household monthly income EUR/1000 (Continuous)** | 0.003 | 0.001 | 1.003 | 1.001-1.005 | *p* < 0.001 |
| **Social activities (with)** | 1.404 | 0.161 | 4.07 | 2.97-5.58 | *p* < 0.001 |

Step 1: Investments in stocks or shares. a: Variable(s) entered on step 1: Social activities (based on 2,901 individuals who answered all the questions).

**7.3.** Social activities and investments in mutual funds or managed investments accounts (controls: gender, age, marital status, number of children and average household monthly income)

| **Variables (Step 1^a^)** | **B** | **S.E.** | **EXP(B) (Odds ratio)** | **95% C.I. for EXP(B)** | **Sig.** |
| --- | --- | --- | --- | --- | --- |
| **Constant** | -2.454 | 0.632 | 0.09 |  | |
| **Gender (male)** | 0.131 | 0.187 | 1.14 | 0.79-1.65 | *p* = 0.483 |
| **Age (Continuous)** | -0.020 | 0.009 | 0.980 | 0.963-0.997 | *p =* 0.024 |
| **Marital status (with relationship)** | 0.422 | 0.185 | 1.53 | 1.06-2.19 | *p =* 0.022 |
| **Number of children (Continuous)** | -0.086 | 0.063 | 0.92 | 0.81-1.04 | *p =* 0.177 |
| **Average household monthly income (EUR/1000) (Continuous)** | 0.002 | 0.001 | 1.002 | 0.999-1.004 | *p* = 0.145 |
| **Social activities (with)** | 1.339 | 0.186 | 3.82 | 2.65-5.49 | *p* < 0.001 |

Step 1: Investments in mutual funds or managed investments accounts. a: Variable(s) entered on step 1: Social activities (based on 2,900 individuals who answered all the questions).

**7.4.** Social activities and investments in at least one risky financial asset (controls: gender, age, marital status, presence of children and average household monthly income)

| **Variables (Step 1^a^)** | **B** | **S.E.** | **EXP(B) (Odds ratio)** | **95% C.I. for EXP(B)** | **Sig.** |
| --- | --- | --- | --- | --- | --- |
| **Constant** | -1.593 | 0.473 | 0.20 |  | |
| **Gender (male)** | 0.209 | 0.140 | 1.23 | 0.94-1.62 | *p* = 0.135 |
| **Age (Continuous)** | -0.022 | 0.007 | 0.98 | 0.97-0.99 | *p* = 0.001 |
| **Marital status (with relationship)** | 0.192 | 0.137 | 1.21 | 0.93-1.59 | *p* = 0.162 |
| **Children (with)** | -0.164 | 0.201 | 0.85 | 0.57-1.26 | *p* = 0.413 |
| **Average household monthly income EUR/1000 (Continuous)** | 0.003 | 0.001 | 1.003 | 1.002-1.005 | *p* < 0.001 |
| **Social activities (with)** | 1.367 | 0.134 | 3.92 | 3.01-5.10 | *p* < 0.001 |

Step 1: Investments in at least one risky financial asset. a: Variable(s) entered on step 1: Social activities (based on 2,892 individuals who answered all the questions).

**8.** **Financial risk-taking intentions and behavior among individuals not participating in social activities, with or without depression caseness:**

| **Not participating in social activities** | | | | | |
| --- | --- | --- | --- | --- | --- |
| **Parameter** | **Asset type** | **Population** | **With and without risk preferences / asset type (N)** | **Willing to take financial risks / with asset type (N)** | **Willing to take financial risks / with asset type (%)** |
| Financial risk-taking intentions | - | Without depression caseness | 8,154 | 1,541 | 18.9 |
|  |  | With depression caseness | 3,669 | 384 | 10.5 |
| Financial risk-taking behavior | Stocks or shares | Without depression caseness | 8,089 | 820 | 10.1 |
|  |  | With depression caseness | 3,738 | 186 | 5.0 |
|  | Mutual funds or managed investments accounts | Without depression caseness | 8,083 | 622 | 7.7 |
|  |  | With depression caseness | 3,733 | 132 | 3.5 |
|  | Both types of risky assets | Without depression caseness | 8,053 | 264 | 3.3 |
|  |  | With depression caseness | 3,723 | 45 | 1.2 |

**References**

1. Mehrbrodt T, Gruber S, Wagner M. Scales and Multi-Item Indicators. SHARE Survey of Health, Ageing and Retirement in Europe. 2021. Available from: https://share-eric.eu/fileadmin/user_upload/Other_Publications/ScalesManual_rel.8-0-0.pdf

2. Mannheim Research Institute for the Economics of Aging. Release Guide 2.3.1. Waves 1 & 2. SHARE Survey of Health, Ageing and Retirement in Europe. 2010. Available from: https://share-eric.eu/fileadmin/user_upload/Bilder_Newsredaktion/SHARE_Release_

Guide_2.3.1.pdf
